# Modified RNA-seq method for microbial community and diversity analysis using rRNA in different types of environmental samples

**DOI:** 10.1371/journal.pone.0186161

**Published:** 2017-10-10

**Authors:** Yong-Wei Yan, Bin Zou, Ting Zhu, Wael N. Hozzein, Zhe-Xue Quan

**Affiliations:** 1 Ministry of Education Key Laboratory for Biodiversity Science and Ecological Engineering, School of Life Sciences, Fudan University, Shanghai, People’s Republic of China; 2 Bioproducts Research Chair, Zoology Department, College of Science, King Saud University, Riyadh, Saudi Arabia; 3 Botany and Microbiology Department, Faculty of Science, Beni-Suef University, Beni-Suef, Egypt; Wageningen University, NETHERLANDS

## Abstract

RNA-seq-based SSU (small subunit) rRNA (ribosomal RNA) analysis has provided a better understanding of potentially active microbial community within environments. However, for RNA-seq library construction, high quantities of purified RNA are typically required. We propose a modified RNA-seq method for SSU rRNA-based microbial community analysis that depends on the direct ligation of a 5’ adaptor to RNA before reverse-transcription. The method requires only a low-input quantity of RNA (10–100 ng) and does not require a DNA removal step. The method was initially tested on three mock communities synthesized with enriched SSU rRNA of archaeal, bacterial and fungal isolates at different ratios, and was subsequently used for environmental samples of high or low biomass. For high-biomass salt-marsh sediments, enriched SSU rRNA and total nucleic acid-derived RNA-seq datasets revealed highly consistent community compositions for all of the SSU rRNA sequences, and as much as 46.4%-59.5% of 16S rRNA sequences were suitable for OTU (operational taxonomic unit)-based community and diversity analyses with complete coverage of V1-V2 regions. OTU-based community structures for the two datasets were also highly consistent with those determined by all of the 16S rRNA reads. For low-biomass samples, total nucleic acid-derived RNA-seq datasets were analyzed, and highly active bacterial taxa were also identified by the OTU-based method, notably including members of the previously underestimated genus *Nitrospira* and phylum *Acidobacteria* in tap water, members of the phylum *Actinobacteria* on a shower curtain, and members of the phylum *Cyanobacteria* on leaf surfaces. More than half of the bacterial 16S rRNA sequences covered the complete region of primer 8F, and non-coverage rates as high as 38.7% were obtained for phylum-unclassified sequences, providing many opportunities to identify novel bacterial taxa. This modified RNA-seq method will provide a better snapshot of diverse microbial communities, most notably by OTU-based analysis, even communities with low-biomass samples.

## Introduction

The amplification of small subunit (SSU) ribosomal RNA (rRNA) genes to study microbial diversity in diverse environments has been performed since the advent of molecular phylogeny. The development of many molecular techniques has aided such studies, such as cloning and sequencing [1], denaturing gradient gel electrophoresis (DGGE) [2], terminal restriction fragment length polymorphism (T-RFLP) analysis [3], and high throughput sequencing [4]. However, these PCR-based techniques largely depend on the coverage of the so-called ‘universal’ primer sets, many drawbacks of which have been reviewed [5–9]. Although metagenomic methods can avoid the bias introduced by PCR-based techniques, they provide very limited 16S rRNA gene related reads in the datasets for analysis [10–13].

The use of RNA-seq in metatranscriptomics provides a snapshot of transcriptional profiles within a microbial community at the time of sampling, providing a feasible method for the functional analysis of microbial communities. However, the target of most metatranscriptomic analyses is mRNA, which only accounts for 1–5% of the total RNA in microbes [14]. Therefore, rRNA is usually removed to enrich mRNA, which is then reverse-transcribed for sequencing and analysis [14–16]. In comparison, rRNA accounts for more than 90% of the total microbial RNA, making rRNA suitable for the analysis of microbial communities. However, mRNA enrichment significantly alters the composition of the remaining microbial rRNA, thus this method cannot be reliably used for community profiling [14]. Urich *et al*. [5] first developed a novel metatranscriptome approach to simultaneously characterize the function and structure of soil microbial community. This ‘Double-RNA approach’, characterizing a microbial community using both rRNA and mRNA, has also been applied in subsequent studies [17–19]. The reverse-transcription of total RNA with random primers can avoid the bias generated by PCR amplification of SSU rRNA gene, and can also compensate for the shortcoming of metagenomics. To enrich SSU rRNA reads in the metatranscriptome, our group developed a gel-extracted SSU rRNA reverse-transcription method for microbial population analysis of active sludge and anaerobic sludge samples [20]. The enriched SSU rRNA-based RNA-seq method has also been applied to the canine mouth [11] and an anaerobic bioreactor [12].

Keeping in mind that a higher potential for protein synthesis would correspond to higher rates of transcribed rRNA including SSU rRNA, the abundance of rRNA for each microbial type, relying on both cell and their ribosome number [12], has been used as an indicator of the potential physiological activity [21]. This methodology offered the advantage of a *priori* knowledge of which main microbial groups and activities were expected [12]. At the same time, it also offers an opportunity to identify potentially active microbes belonging to the ‘shadow biosphere’ that have escaped detection due to the inefficient coverage of ‘universal’ primer sets [22].

However, microbial population analysis utilizing SSU rRNA within RNA-seq data has not been broadly applied in the field of microbial ecology because its accuracy has not been confirmed using a mock community. Additional reasons for the limited application of this method include the requirement for high qualities of RNA [11] and the difficulty of performing alpha- and beta-diversity analyses due to differences in the regions covered by the sequences, although we already used V3 region covered sequences (32–36% of the reverse-transcribed SSU rRNA sequences) in a previous study [20].

In this study, a modified method is presented to construct a transcriptome library using a low quantity of RNA that is suitable for SSU rRNA sequencing and analysis. The developed method has several advantages, and its accuracy was confirmed using mock communities. In addition, non-coverage rates of a universal primer were evaluated within different bacterial taxa.

## Materials and methods

### Ethics statement

For the environmental samples taken from Chongming Island, such as salt-marsh sediments and mudflat surface water, sampling activities were permitted by the Chongming Dongtan National Nature Reserve Management Office, Shanghai, China. For microbial sampling of tap water and the leaf surfaces of *Osmanthus fragrans*, no specific permissions were required since they were of publicly available and our activities did not have any effects on their normal use. All field studies did not involve endangered or protected species. Sampling of forehead skin surfaces was approved by the Ethical Committee of Fudan University as a part of a human skin-related study prior to implementation.

### Description of samples

For mock communities, three bacterial isolates (*Bacillus subtilis* WB600, *Chryseobacterium caeni* N4^T^, and *Escherichia coli* DH5α), one archaeal isolate (*Halobacterium halobium* CCTCC AB 91027) and one fungal isolate (*Pichia pastoris* CS115) were used in this study.

Tidal salt-marsh sediments (0–2 cm) were collected from two sites in Dongtan of Chongming Island in November, 2014 as described previously [23], with each site vegetated by *Phragmites australis* (Pa) and *Spartina alterniflora* (Sa), respectively. Mudflat surface water was collected from Chongming Island (121°57′E, 31°33′N) in October, 2015. All Chongming Island samples were collected in triplicate and pooled, then were transferred to the lab on ice within two hours. Mudflat surface water (300 mL) was filtered through 25 mm diameter, 0.22 μm mixed cellulose ester (MCE) filters (Sangon Biotech, Shanghai, China), taking approximately 5 minutes. A total volume of 500 mL of tap water was used for filtration using the same method. Microbes from a shower curtain were collected in a male dormitory of Fudan University in November, 2015. Microbes were also sampled from the surface of *Osmanthus fragrans* leaves that were collected from Fudan University campus, and those sampled from foreheads were collected from three adult volunteers. All surface samples were collected in triplicates using swabs, with each collection taken from a 25 cm^2^ area (for the leaf surfaces a 10 cm^2^ area was used) with polyester fiber-tipped swabs that were moistened with a solution of 0.15 M NaCl and 0.1% Tween 20. The swab heads were removed using sterilized tweezers and were carefully stored in Lysis/Binding buffer (Ambion, Austin, TX) at 4°C until RNA extraction.

### RNA extraction

Total RNA of all prokaryotes was isolated using RNAiso Plus reagent (TaKaRa, Dalian, China) after a lysozyme digestion procedure. Fungal RNA was extracted as described by Mannan *et al*. [24]. Total nucleic acid (NA) was extracted from 0.5 g of salt-marsh sediments as recommended by Lüdemann *et al*. [25]. Isolation of total NA from the remaining five samples was conducted using a mirVana RNA Isolation Kit (Ambion, Austin, TX) according to the manufacturer’s instructions after a glass beads vortex step.

The total RNA or NA was visualized in a 1% (w/v) agarose gel after electrophoresis to assess the sample integrity. The bands containing SSU rRNA of microbial isolates and Pa sediments were excised from the agarose gel, and SSU rRNA was then purified from the gel using a Qiaquick Gel Extraction Kit (Qiagen, Hilden, Germany). Electrophoresis was conducted to assess the integrity of enriched SSU rRNA, and a BioAnalyzer 2100 (Agilent Technologies, Palo Alto, CA) was used to check for any contamination.

Total NA from all samples was stored at -80°C and RNA samples were quantified using a Qubit RNA Assay Kit on Qubit 2.0 fluorometer (Life Technologies, Carlsbad, CA) before the preparation of RNA-seq libraries.

### Preparation of RNA-seq libraries

All RNA-seq libraries in our study were prepared using a method that was modified from the RNA-seq Library Preparation Kit protocol (Gnomegen, San Diego, CA). Total NA or gel-extracted SSU rRNA was heat-denatured at 65°C for 5 minutes instead of the kit-suggested fragmentation at 95°C. Next, a RNA-seq 5’ adaptor was directly ligated to the 5’ end of the heat-denatured full-length RNA at 37°C for two hours. After the ligation products were purified using a Gnome Size Selector (Gnomegen, San Diego, CA), the first strand of cDNA was synthesized from the products with a tagged random hexamer. The cDNA was also purified using a Gnome Size Selector according to the standard instructions. To enrich the products for sequencing, 15-cycles of PCR amplification were performed on the first cDNA strands using Illumina compatible primer sets. These primers were designed according to the adaptor and tag sequences and were complementary to the standard Illumina forward and reverse primers. The reverse primer also contained an 8-nucleotide (nt) indexing sequence to allow for multiplexing. The 400–600 base pair (bp) PCR products were size-selected using a Gnome Size Selector and were sequenced on an Illumina MiSeq platform using the 2 x 300 paired end protocol.

A preliminary experiment was designed to confirm that genomic DNA would not interfere with the library preparation and bioinformatics analysis. Total NA from Sa sediments were digested with DNase I (TaKaRa, Dalian, China) for one hour, and total RNA was purified with a MiniElute Cleanup Kit (Qiagen, Hilden, Germany). The absence of residual genomic DNA was assessed by a 30-cycle PCR amplification of the purified RNA with the bacterial universal primers 8F and 536R [8]. Next, 25 or 200 ng of λ phage DNA were respectively added into two sets of 25 ng purified total RNA, and RNA-seq libraries were also constructed as described above. Products of the libraries were then A-tailed and cloned into pMD19-T vectors (TaKaRa, Dalian, China) for sequencing of positive clones to detect λ phage sequence-related clones.

To test the accuracy of the SSU rRNA-based community analysis using the modified RNA-seq method, three mock communities (Mock 1, 2 and 3) were constructed by combining SSU rRNA of the five microbial isolates at different ratios (S1 Table). For environmental samples, the RNA quantities used as input for RNA-seq library construction are listed in S2 Table. The steps used to prepare all libraries were performed as described above. The RNA-seq library construction with or without RNA denaturation was also performed on mock communities to determine the effect on community structures.

### Analysis pipeline

Paired-end reads were pre-processed with Sickle software v1.33 [26] to trim and filter reads with a phred quality score below 20. *De novo* assembly was conducted with the command join_pair_end.py in QIIME v1.8.0 [27]. Assembled sequences with ambiguous nucleotides and homopolymer lengths longer than eight nucleotides were removed in mothur v1.33.3 [28].

For taxonomic annotation of mock communities, a SILVA-formatted SSU rRNA reference database was constructed based on nearly complete SSU rRNA sequences of the five isolates (GenBank accession numbers CP015004, DQ336714, CP014092, M11583 and FR839631). SILVA-formatted LSU rRNA (large subunit rRNA) reference database was constructed with the same method by using the complete LSU rRNA sequences (GenBank accession numbers CP009749, AUFK01000006, AE014075, X03407 and FN392325). The ‘minlength’ parameter of the ‘screen.seqs’ command was set several times to select the appropriate size of sequences for classification. Taxonomic annotations were determined using the ‘classify.seqs’ command in mothur v1.33.3 with a bootstrap cut-off of 80% as previously proposed [29].

To correctly assign all SSU rRNA reads within the RNA-seq datasets of the environmental samples, sequences longer than 250 bp were classified against the SILVA SSURef v119 database in MIPE (https://github.com/zoubinok/MIPE) with a bootstrap cut-off of 80%, and sequences that were identified as chloroplast, mitochondria or human were removed. Mismatches of the utilized primer 8F (5’- AGAGTTTGAT (C/T) (A/C) TGGCTCAG-3’) [8] in all the bacterial SSU rRNA sequences were also identified in this software. Candidate LSU rRNA sequences were determined by their maximum alignment scores from the output of four alignment files and were annotated using the SILVA LSUref v119 database in mothur v1.33.3. Sequences that fell below a cut-off threshold of less than 80% for the SSU and LSU rRNA annotations or which had an alignment score lower than 10 were considered to be non-rRNA sequences and were aligned to the NCBI non-redundant protein database (released on November 26, 2015) using BLASTX with an e value of 10^−5^ to identify mRNA transcripts. Identified archaeal and bacterial SSU rRNA sequences were aligned and trimmed to leave the 8F-V1-V2 region (*E*. *coli* position 8 to 242), and the OTU (operational taxonomic unit) based community structure and diversity index calculations were analyzed as described by Kozich *et al*. [30]. Representative OTU sequences at a cut-off of 0.03 were taxonomically classified against the SILVA SSURef v119 database with a bootstrap cut-off of 50% as previously recommended [29], and OTUs belonging to chloroplasts or mitochondria were also removed from the analysis. For datasets of tap water, shower curtain, leaf surfaces, mudflat surface water and forehead, SSU rRNA sequences of the prevalent fungal phylum were aligned against the SILVA SSU rRNA database for detailed taxonomic information using BLASTn with an e value of 10^−5^. The sequence datasets of the environmental samples generated in this study were deposited in the NCBI GenBank Short Read Archive (SRA) under the accession numbers SRR5172053- SRR5172059.

### Statistical analysis

Linear regression analyses were performed to compare community compositions between RNA-seq and mock datasets for the three mock communities, and community compositions of Pa sediments derived from different methods. A two-tailed Fisher’s exact test was performed in STAMP software [31] to determine the significance of difference in proportions for each sequence type between the two Pa sediment datasets.

## Results

### Accuracy test

**Fig 1:**
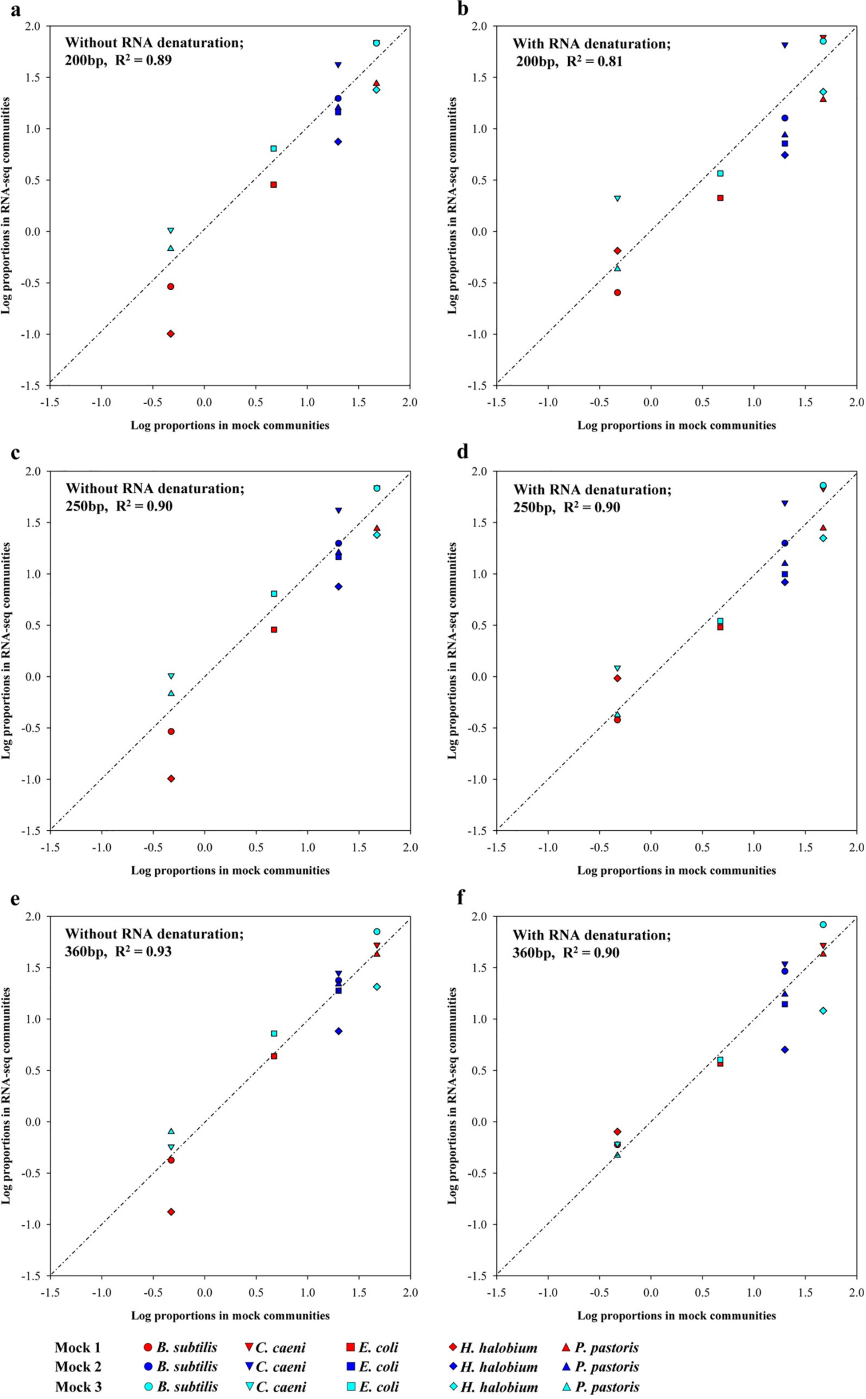
Comparison of the communities identified by SSU rRNA between RNA-seq and mock datasets. The percentages of SSU rRNA sequences for a particular strain within the two datasets were plotted. The 200, 250 and 360 bp values in the legends represent the trimmed minimum sequence lengths used for the community analysis. R^2^ values are coefficients of the regression equations.

Using a minimum input of 10 ng of SSU rRNA for library construction (S1 Table), three mock communities, containing bacterial, archaeal and fungal SSU rRNA were analyzed using more than 30 thousand SSU rRNA reads for each community (S3 Table). Overall, the communities that were determined using the RNA-seq datasets were highly consistent with the mocks according to the correlation analysis (r^2^ = 0.81–0.93) (Fig 1). Moreover, the detected relative contents of the bacterial isolates *B*. *subtilis* and *E*. *coli*, and the yeast isolate *P*. *pastoris* matched with those present in the theoretical mock communities, while the bacterial isolate *C*. *caeni* and the archaeal isolate *H*. *halobium* were over or under estimated (S4 Table). Without heat-denaturation of the mixed SSU rRNA prior to the adaptor ligation step, a sequence length-dependent ratio variation for *C*. *caeni* and *H*. *halobium* was observed, and the effect became more noticeable for two sequence length cut-off values (>200 bp and >360 bp). When the minimum length was at 200 bp, the *C*. *caeni* contents were consistently overestimated by approximately two to threefold, in contrast to the two to threefold underestimation observed for *H*. *halobium*. If sequences longer than 360 bp were utilized, all the ratios were much closer to the theoretical ones except for *H*. *halobium*. To decrease the effect of RNA secondary structure on reverse-transcription, a denaturation step for mixed SSU rRNA was added before the adaptor ligation step, and the three communities were re-examined. Although the RNA denaturation increased the *C*. *caeni* bias, the underestimation bias for *H*. *halobium* was decreased, and the *C*. *caeni* bias could also be controlled by the selection of a cut-off sequence length (>250 bp).

**Fig 2:**
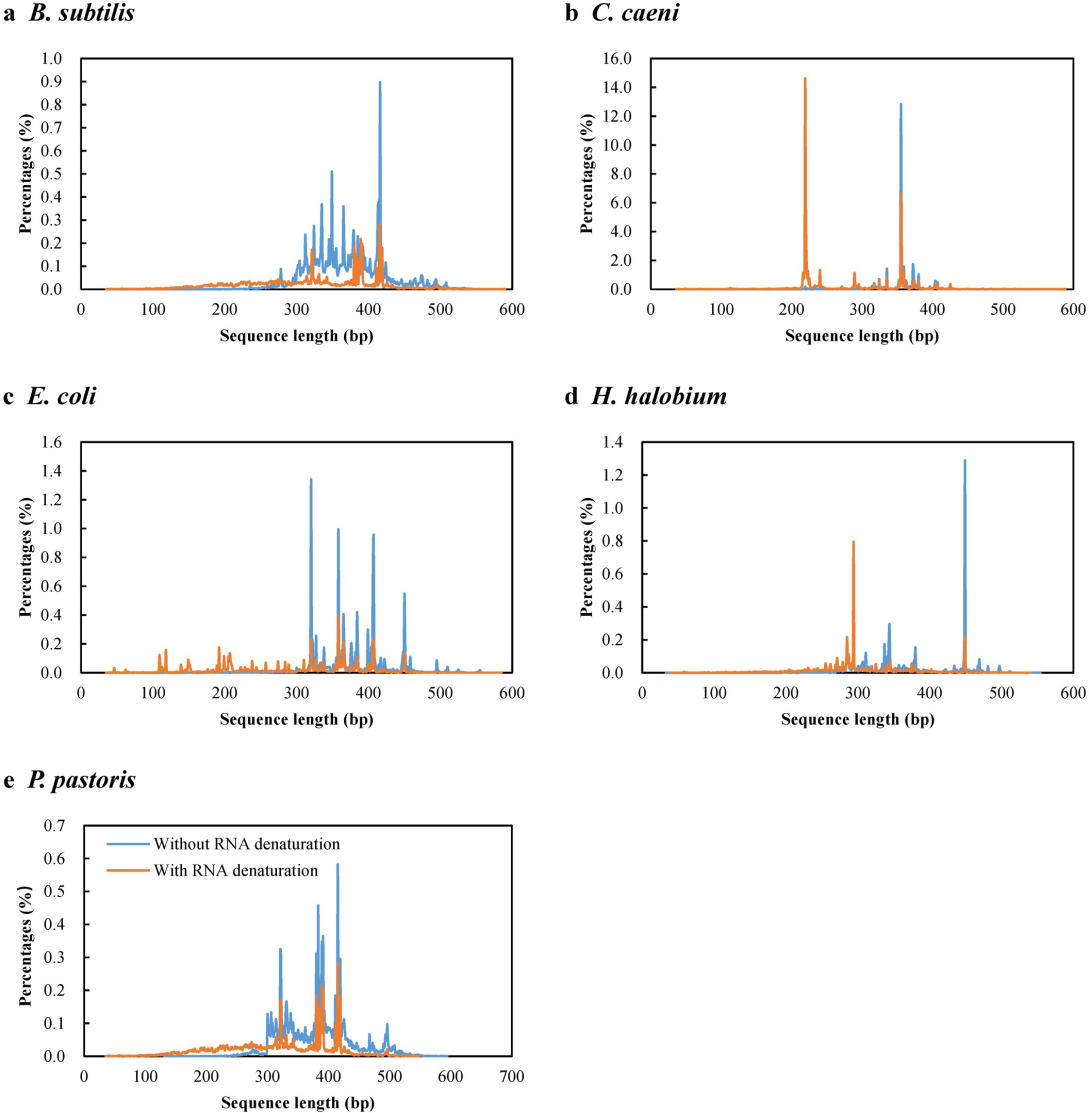
Length distribution of SSU rRNA sequences for the five microbial isolates in the Mock 2 community. A comparison was conducted between different RNA processing methods, without (blue) and with (orange) RNA denaturation. The x-axis indicates the read length of the SSU rRNA sequences, and the y-axis indicates the percentages of the SSU rRNA sequences obtained for the five isolates.

The Mock 2 community was selected for the analysis of size distribution patterns of the five isolates since their SSU rRNA were equally abundant in the community. In Fig 2, it can be clearly observed that their distribution patterns were different, with several unique peaks within each isolate. However, some unique peaks for each isolate overlapped with or without the initial RNA denaturation process. Extremely high numbers of peaks in the sequence length distribution of *C*. *caeni* and *H*. *halobium* were observed, which sharpened at 219 bp (0.2 and 14.6%) and 355 bp (12.8 and 6.6%) for *C*. *caeni* and at 294 bp (0.5 and 0.8%) and 449 bp (1.2 and 0.2%) for *H*. *halobium*. During the construction of the library, some amplification cycles were required before enough double-stranded cDNA could be obtained for sequencing. Because the PCR amplification efficiency is much higher for shorter size sequences [32], the short cut-off length (200 bp) used for the RNA denatured mock communities notably increased the overestimation of *C*. *caeni*. In contrast, the long cut-off length (360 bp) caused the proportion of *H*. *halobium* to decrease because the primary unique length sequences were discarded. Therefore, the 250 bp cut-off length was selected for the following analysis.

### Sequence type

In the preliminary experiments, even when high amounts of λ phage DNA was added into purified total RNA before RNA-seq library preparation, no λ phage sequences were detected within the library products (data not shown). This confirmed the hypothesis that residual genomic DNA has no effect on the library construction and analysis. Therefore, the RNA-seq libraries of all the environmental samples, in addition to an SSU rRNA enriched library for Pa sediments, were constructed from total NA.

Raw sequences in all the RNA-seq data were trimmed, assembled and filtered by quality and lengths. For RNA-seq datasets of mock communities and environmental samples, the sequence information retained after these preprocesses is shown in S3 Table and Table 1, respectively.

**Table 1:** Contents of different sequence types within RNA-seq datasets of environmental samples.

		Pa-Total ^a^		Pa-SSU ^b^		Tap Water (TW)		Shower Curtain (SC)		Leaf Surfaces (LS)		Mudflat Water (MW)		Forehead (FH)	
Sequence type		Counts	%	Counts	%	Counts	%	Counts	%	Counts	%	Counts	%	Counts	%
**All**		**406,164**		**426,311**		**205,258**		**336,092**		**484,020**		**168,730**		**255,433**	
**LSU rRNA**		**109,497**	**27.0**	**114,257**	**26.8**	**126,280**	**61.5**	**239,948**	**71.4**	**412,872**	**85.3**	**128,881**	**76.4**	**162,377**	**63.6**
	Archaea	1,936	1.8	1,921	1.7	8	0.0	11	0.0	21	0.0	36	0.0	129	0.1
	Bacteria	66,235	60.5	78,362	68.6	53,462	42.3	64,054	26.7	13,546	3.3	8,981	7.0	91,545	56.4
	Eukaryota	41,326	37.7	33,974	29.7	72,810	57.7	175,883	73.3	399,305	96.7	119,864	93.0	70,703	43.5
**SSU rRNA**		**220,427**	**54.3**	**277,826**	**65.2**	**65,655**	**32.0**	**91,976**	**27.4**	**65,530**	**13.5**	**33,514**	**19.9**	**82,945**	**32.5**
	Archaea	3,383	1.5	4,027	1.4	13	0.0	14	0.0	23	0.0	43	0.1	159	0.2
	Bacteria	159,684	72.4	26,2015	94.3	51,198	78.0	70,878	77.1	16,254	24.8	9,542	28.5	62,870	75.8
	Eukaryota	57,360	26.0	11,784	4.2	14,444	22.0	21,084	22.9	49,253	75.2	23,929	71.4	19,916	24.0
**mRNA**		**8,191**	**2.0**	**6,863**	**1.6**	**8,939**	**4.4**	**1,311**	**0.4**	**2,264**	**0.5**	**2,195**	**1.3**	**2,116**	**0.8**
**Others** ^**c**^		**68,049**	**16.8**	**27,365**	**6.4**	**4,164**	**2.0**	**2,759**	**0.8**	**3,354**	**0.7**	**4,140**	**2.5**	**7,461**	**2.9**

^a^ RNA-seq dataset of salt-marsh sediments vegetated by *Phragmites australis*, for which total nucleic acid was used for library preparation.

^b^ RNA-seq dataset of salt-marsh sediments vegetated by *Phragmites australis*, for which only enriched SSU rRNA was used for library preparation.

^c^ Unassigned sequences and sequences belonging to chloroplasts, mitochondria or human.

Most of the classified sequences (74.9–96.4%) in mock communities belonged to SSU rRNA of the five isolates (S3 Table). However, a small fraction of LSU rRNA sequences still remained in these enriched SSU rRNA-derived datasets, accounting for 2.6 to 12.5% of the total sequences in these groups (S3 Table). For RNA-seq datasets of environmental samples, more than 80% of the sequences were rRNA sequences (Table 1). For Pa sediments, the difference in SSU rRNA sequences within the two datasets was only approximately 10%, although the proportion in the enriched SSU rRNA-derived dataset (Pa-SSU) was significantly increased compared with that observed in the dataset derived from total NA (Pa-Total) (Fisher’s exact test, *P* < 0.001). In addition, no significant difference was observed for the fraction of LSU rRNA sequences between the two datasets (Fisher’s exact test, *P* > 0.05). The proportion of *Eukaryota* in SSU rRNA reads of Pa-SSU (4.2%) was significantly decreased compared with that in Pa-Total (26.0%) (Fisher’s exact test, *P* < 0.001), which may be attributed to the loss of some long sequences related to eukaryotic 18S rRNA during gel-purification of SSU rRNA.

### Position distribution of 16S rRNA and non-coverage evaluation of the 8F primer

**Fig 3:**
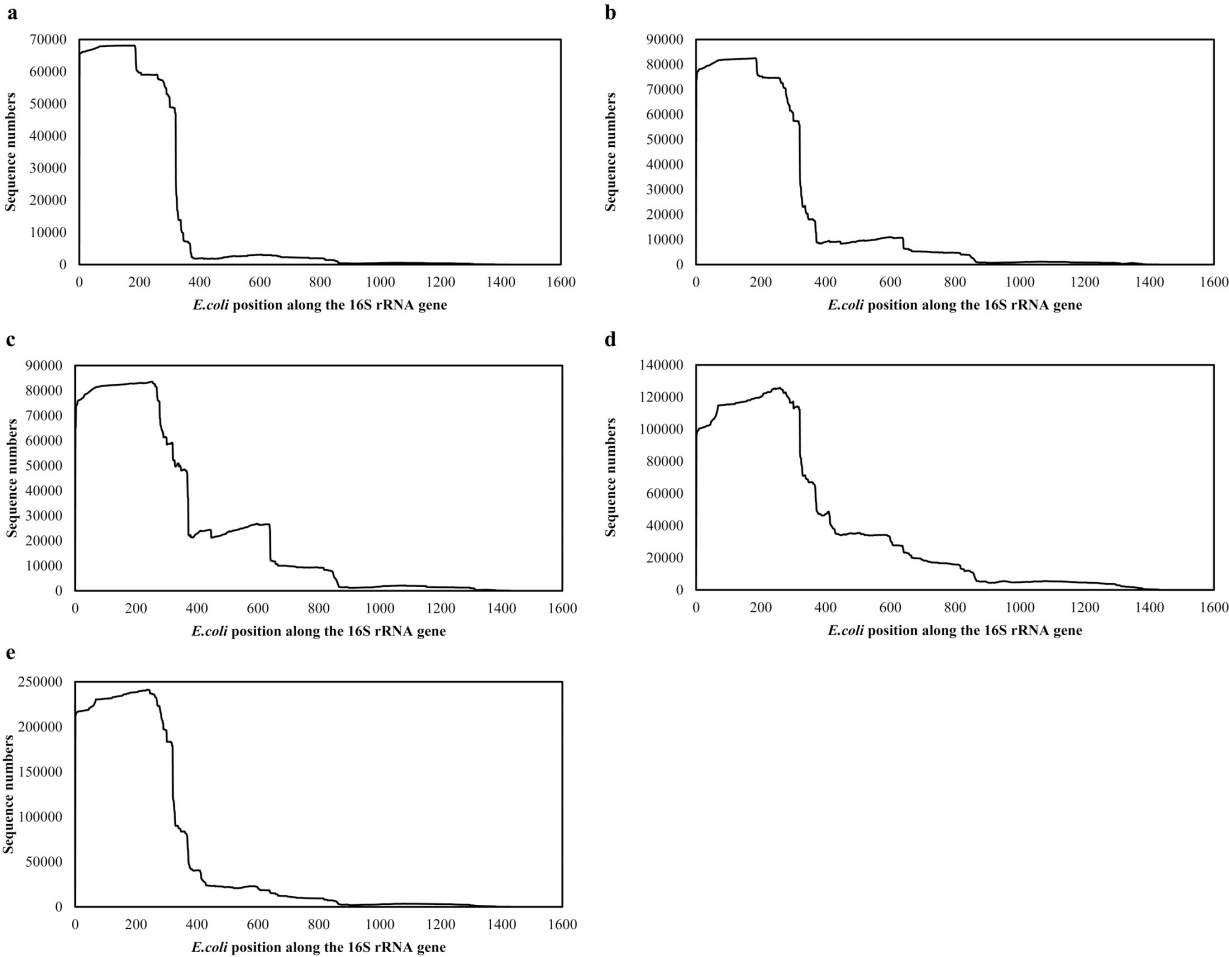
Position distribution patterns of 16S rRNA sequences from different RNA-seq datasets. An RNA denaturation step was performed before library preparation. a, Mock 1; b, Mock 2; c, Mock 3; d, Pa-Total; e, Pa-SSU. Explanations for Pa-Total and Pa-SSU are given in Table 1.

The 16S rRNA sequences longer than 250 bp were selected from the mock community datasets to analyze position distributions (Fig 3A–3C). The distributions of prokaryotic 16S rRNA from these communities were similar and primarily covered the 8F primer, V1 and V2 regions between *E*. *coli* positions 8 to 242 (78.7, 72.1, and 65.7% for the three mock communities, respectively). For the 16S rRNA present within the datasets, such as for Pa-Total and Pa-SSU (46.4 and 59.5% respectively), the distribution patterns were similar to those of the mock communities (Fig 3D and 3E). Further analysis confirmed the percentages of bacterial 16S rRNA sequences in OTU-related community analysis, ranging from 44.5%– 59.8% (S7 Table).

**Fig 4:**
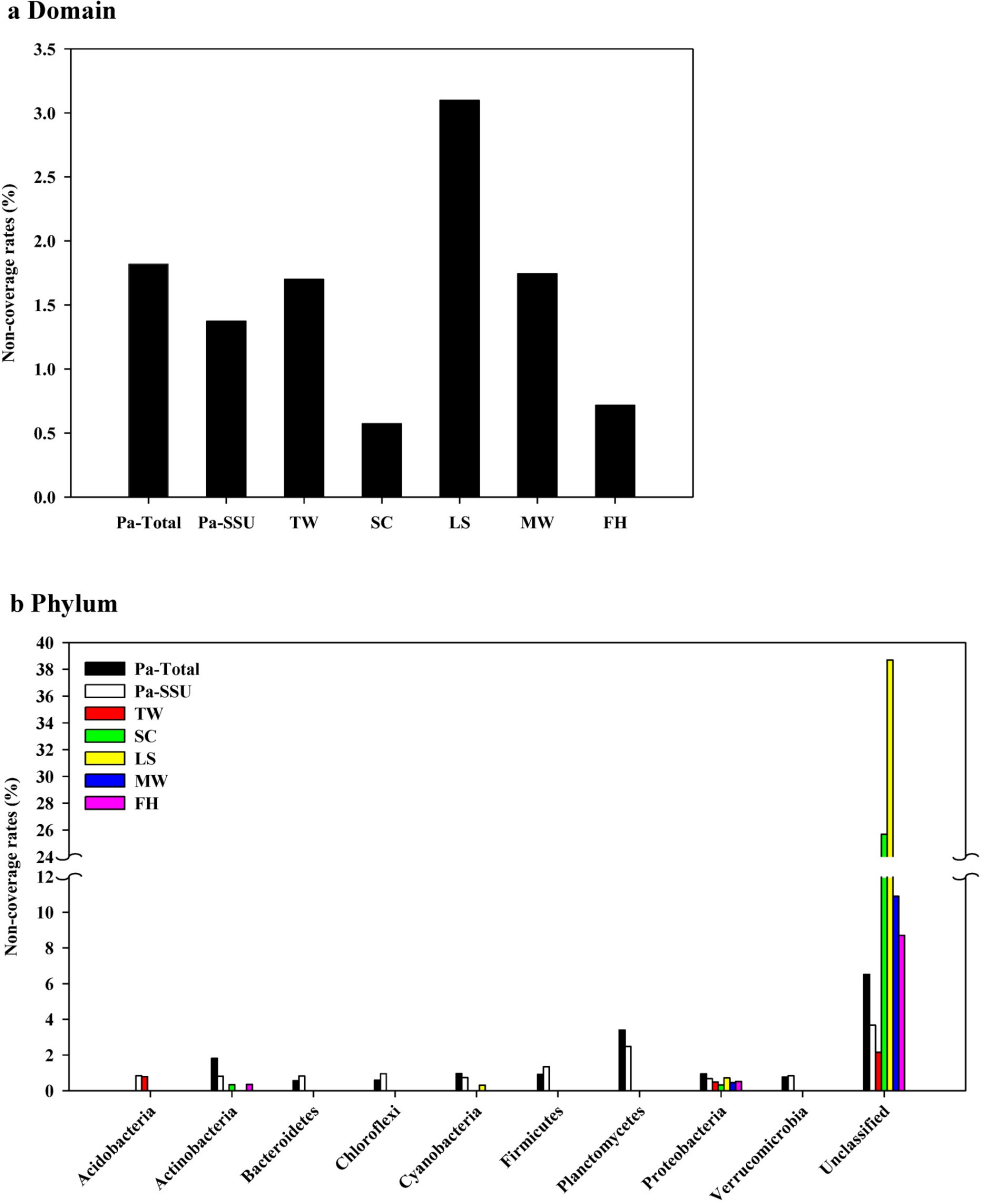
Non-coverage rates of the bacterial primer 8F. Evaluation of non-coverage rates for primer 8F in total bacteria (a) and different phyla (b). The phyla with less than 10 non-coverage sequences in the datasets are not shown. The non-coverage rates were calculated by dividing non-coverage sequences (having at least one mismatch within primer 8F) of taxa with their relative total sequences. Explanations of abbreviations are given in Table 1.

More than half of the bacterial 16S rRNA sequences in each dataset were observed to contain the complete 8F primer region, and non-coverage rates of the primer were evaluated at different taxonomic levels by using all these SSU rRNA sequences (S5 Table). Only a small proportion of the detected bacterial reads contained mismatched nucleotides, accounting for just 0.57 and 3.10% of the reads in the shower curtain and leaf surfaces datasets, respectively (Fig 4A). Such sequences were distributed among various phyla, with those of the phylum *Proteobacteria* discovered in all datasets and had less than 1% non-coverage rates (Fig 4B). However, for the phylum-unclassified sequences, mismatch rates of up to 38.7% were observed (leaf surfaces dataset), much higher than that of other phyla (Fig 4B, S5 Table).

### Community structures and diversity analysis for environmental samples

Both Pa sediment samples and some low-biomass samples from diverse environments, such as tap water, shower curtain, leaf surfaces, mudflat surface water and the skin of human foreheads, were utilized for analysis of community structures with the modified RNA-seq method described in this study.

**Fig 5:**
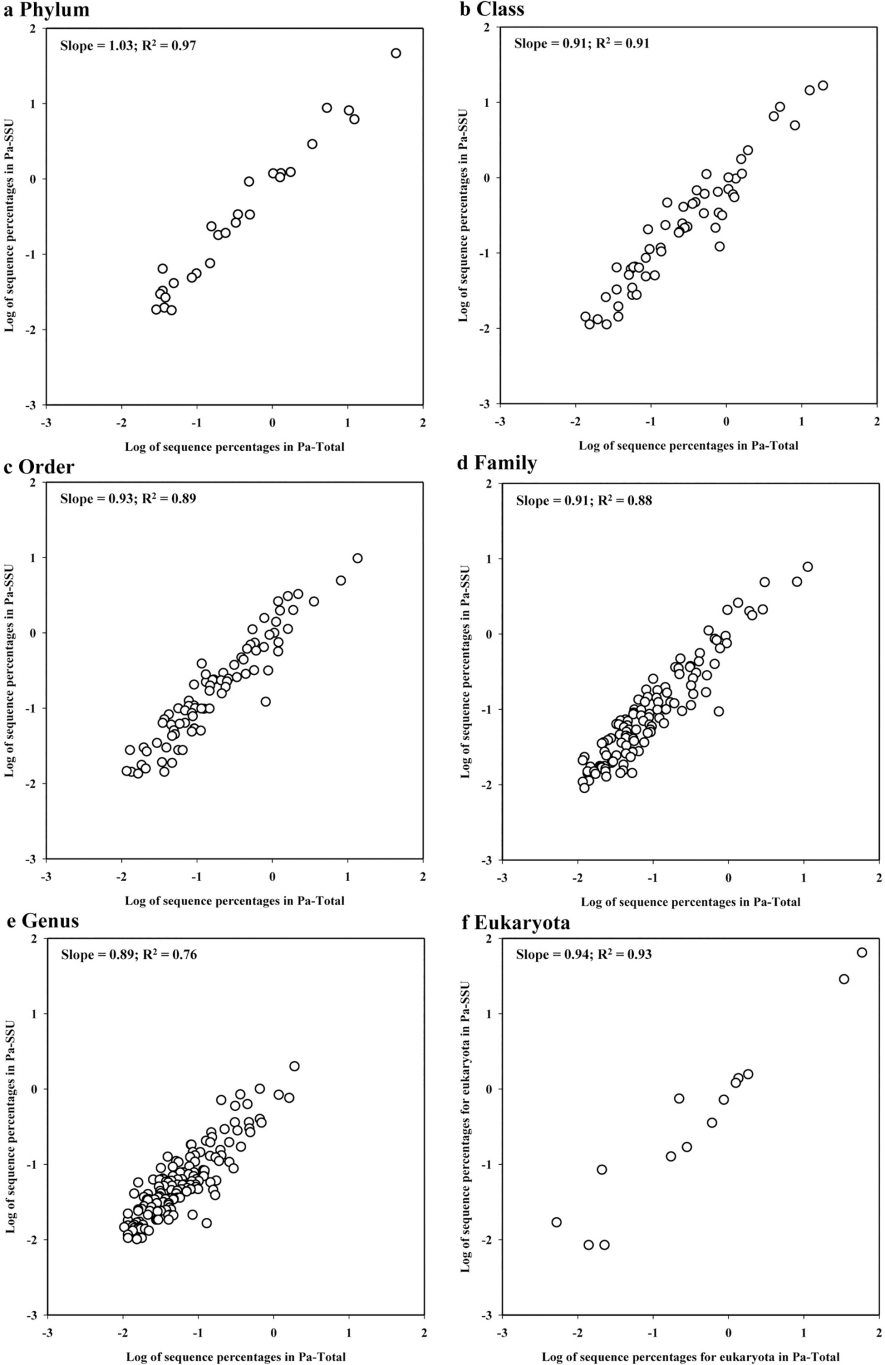
Comparison of the communities identified by SSU rRNA between enriched SSU rRNA- and total NA-derived RNA-seq datasets at different taxonomic levels. a, Phylum; b, Class; c, Order; d, Family; e, Genus; and f, Eukaryota. The percentages of SSU rRNA sequences for a particular taxon within the two datasets were plotted, and the eukaryotic percentages were plotted according to relative abundances of the fourth rank based on the SILVA database. The values in the legends are the slopes and coefficients of the regression equations, respectively. Explanations of Pa-Total and Pa-SSU are given in Table 1.

Community structures of Pa sediments were initially characterized using all SSU rRNA sequences from the Pa-Total and Pa-SSU datasets. The correlation between the community structures was studied and the percentages of each specific taxon were plotted at different taxonomic levels (Fig 5). The microbial communities were very similar to each other, even at genus level (slopes of the regression equations were 0.89 to 1.03, r^2^ > 0.76), although all SSU rRNA sequences had very different community proportions between the Pa-Total and Pa-SSU at the domain level, notably for *Eukaryota* (26.0% *vs*. 4.2%). In addition, a good correlation was also observed in their eukaryotic communities (r^2^ = 0.93). OTU-based prokaryotic structures were also described for the two datasets using only the 8F-V1-V2 containing 16S rRNA sequences. Such community structures also correlated well with those determined by all the SSU rRNA sequences above the genus level (slopes of 1.00–1.08 and r^2^ > 0.84 for Pa-Total; slopes of 0.91–1.00 and r^2^ > 0.81 for Pa-SSU) (S1 and S2 Figs).

**Fig 6:**
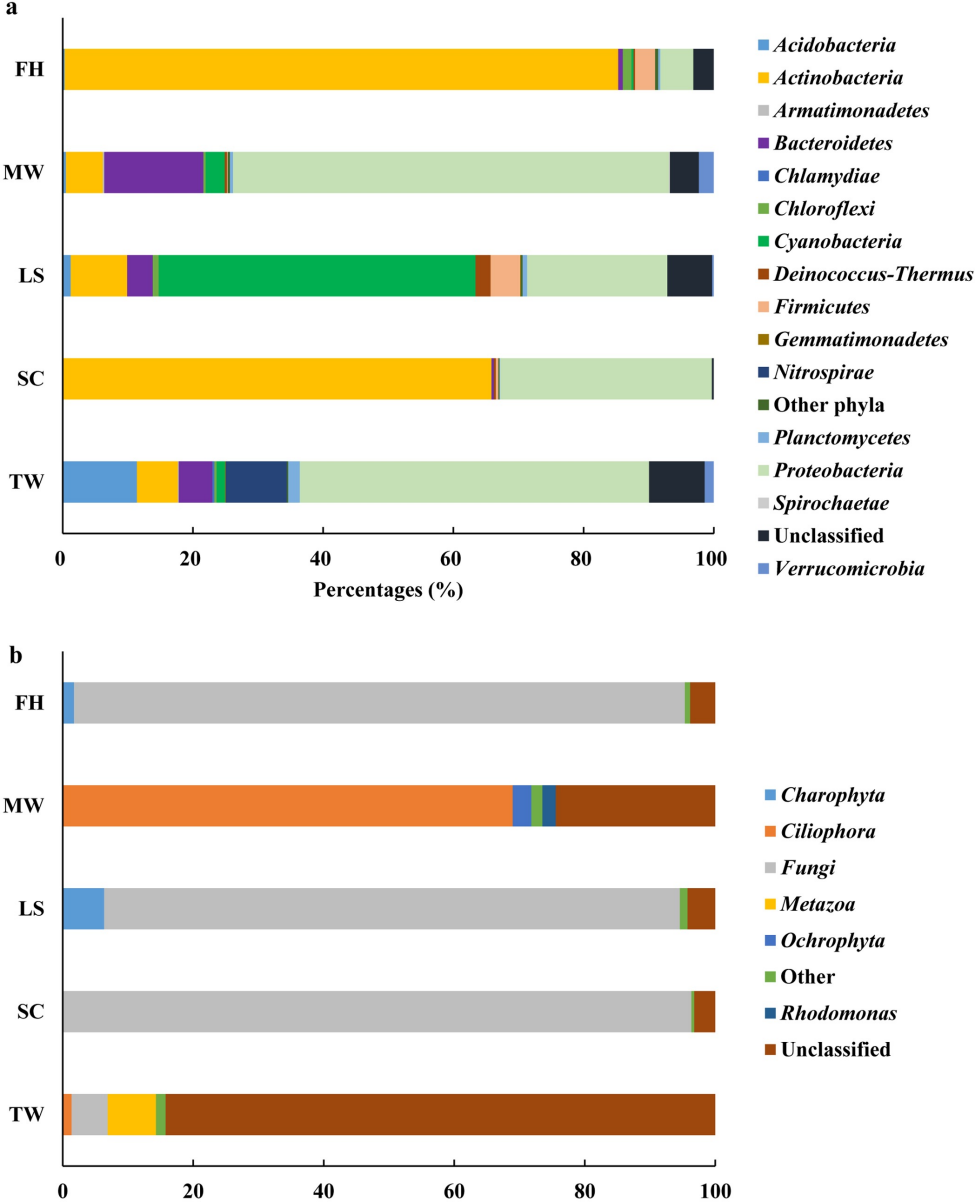
Relative abundances of bacterial and eukaryotic taxa in the five datasets. a, relative abundance of bacterial taxa at phylum level; b, relative abundance of representative eukaryotic taxa at the fourth rank. ‘Other phyla’ or ‘Other’, includes taxa that made up of small fractions (<1%). ‘Unclassified’, includes sequences under a bootstrap cut-off value of 50% for bacteria or 80% for eukaryota. Explanations of abbreviations are given in Table 1.

SSU rRNA sequences of the other five datasets were also analyzed, as mentioned above. At the domain level, *Eukaryota* dominated the SSU rRNA sequences of the leaf surfaces (75.2%) and mudflat surface water (71.4%) (Table 1). In contrast, *Bacteria* dominated SSU rRNA sequences in the other three datasets, and the proportions ranged from 72.1 to 78.0% (Table 1). Because of the small proportions of *Archaea*, only *Bacteria* and *Eukaryota* were selected for further analysis. OTU-based communities were determined for bacteria (Fig 6A, S6 Table), while eukaryotic communities were profiled using all their 18S rRNA sequences (Fig 6B, S6 Table).

At the phylum level, *Proteobacteria* or *Actinobacteria* was the most abundant potentially active taxa in all bacterial communities of low-biomass samples, except that of leaf surfaces. For example, the phylum *Proteobacteria* predominated in tap water (53.64%) and mudflat surface water (66.96%) datasets, while *Actinobacteria* predominated the shower curtain (63.42%) and forehead (81.80%) datasets. In *Proteobacteria* community of tap water, the *α-* (21.30%) and *β-* (24.17%) *Proteobacterial* classes were prevalent, with more than half of their sequences belonging to the order *Rhizobiales* and the family *Comamonadaceae*, respectively. In comparison, the most potentially active *Proteobacterial* taxa within mudflat surface water were determined to be in the family *Rhodobacteraceae* in the class *Alphaproteobacteria*, the family *Comamonadaceae* in the class *Betaproteobacteria* and the order *Alteromonadales* in the class *Gammaproteobacteria*. In addition to *Proteobacteria*, two more abundant potentially active taxa in tap water were assigned to the class *Acidobacteria* in the phylum *Acidobacteria* (11.39%) and to the genus *Nitrospira* in the phylum *Nitrospirae* (9.39%), compared to the only other potentially active phylum *Bacteroidetes* (15.24%) present in mudflat surface water. For the potentially active phylum *Actinobacteria*, the prevalent members within the shower curtain dataset were the families *Intrasporangiaceae* (22.5%), *Micrococcaceae* (19.0%) and *Nocardiaceae* (12.3%), different from the absolute single dominance of the genus *Propionibacterium* (80.47%) observed in forehead dataset. For the leaf surfaces, the potentially active bacteria were dominated by four subsections of phylum *Cyanobacteria* (48.6%), notably the genus *Chroococcidiopsis* of subsection II, although *Proteobacteria* (21.5%) and *Actionobacteria* (8.7%) were also relatively abundant.

In potentially active eukaryotic communities, *Fungi* was the most abundant in the shower curtain, leaf surfaces and forehead, in contrast to the small fraction it accounted for in tap water. Although the phylum *Ascomycota* predominated in shower curtain and leaf surfaces datasets (96.4 and 88.2%, respectively), nearly all the fungal sequences within the shower curtain dataset belonged to the classes of *Dothideomycetes* Incertae sedis and *Dothideomycetes* contrasting to the predominance of orders *Dothideales* and *Pleosporales* in leaf surfaces. For forehead, phylum *Basidiomycota* accounted for 85.7% and nearly all the sequences were assigned to the genus *Malassezia*. The most abundant potentially active eukaryotic taxa in mudflat surface water were classified as the protozoan *Ciliophora*, though approximately one fourth of the 18S rRNA sequences in this dataset were unclassified.

The alpha diversity indices of the environmental samples were calculated by employing 8F-V1-V2 containing sequences at different cut-off values (S7 Table), and comparisons were conducted when same number of sequences were subsampled (S3 Fig). For Pa sediments, the obtained number of OTUs for Pa-Total was approximately equal to that for Pa-SSU at any cut-off distance for archaea or bacteria. The same trend was also observed from the Chao, ACE and Shannon analyses. In the other five datasets, the leaf surfaces showed relatively high diversity.

## Discussion

Since the first use of next-generation sequencing in metatranscriptomics, the use of RNA-seq approaches has provided insights into the function of microorganisms in diverse environmental samples. However, for RNA-seq analyses, a high input quantity (>100 ng) of purified RNA is usually required [15, 16, 33, 34], while SSU rRNA enriched method requires even higher amounts [11, 20]. Because adaptor ligation generally occurs after random-primed double-stranded cDNA synthesis in standard protocols [35], contaminated DNA should be removed before library preparation. In the present study, a modified method of using a low input (10–100 ng) of RNA by combining a 5’ end adaptor pre-ligation for library construction was developed to remove the interference of contaminating DNA. In addition to an accuracy analysis in mock communities in this study, different types of environmental samples (Pa sediment samples, and low-biomass samples, such as tap water) were also selected to evaluate the developed method.

### Experimental and analytical reliability of the RNA-seq method

The reliability and accuracy of the developed method was confirmed by the composition analysis of three mock communities, which included SSU rRNA of microbial isolates from the three domains of life, *Archaea*, *Bacteria*, and *Eukaryota*. The populations could be determined when the SSU rRNA was mixed at a span of 0.1 to 10 fold, though the ratios of *C*. *caeni* and *H*. *halobium* showed two or three folds bias. To improve the accuracy of this method, heat-denaturation of RNA before the adaptor ligation step was proposed. The results of the size distribution patterns of the assembled sequences clearly showed that the patterns of different microbes were different, with several unique peaks being present for each isolate, suggesting that the hybridization of random primers to RNA was not randomized [35, 36] and that the sequence length used for the community analysis is also a factor that should be taken into account. In addition, the adaptor ligation efficiency [37] and PCR amplification [38] of templates with different GC contents during library construction of RNA-seq can also bias the content estimation of *H*. *halobium* (68% GC content in 16S rRNA) and *C*. *caeni* (34% GC content in 16S rRNA).

The bacterial or fungal composition and calculated diversity indices of Pa-SSU correlated well with that of Pa-Total. However, it is notable that the content of the remaining LSU rRNA sequences in Pa-SSU was highly consistent with a previous report [20]. These LSU rRNA sequences may have originated from self-primed synthesis [39] or a physical shearing effect during RNA extraction [40]. A closer inspection for LSU rRNA sequences of the five isolates in Mock 2 community revealed that the starting points of adaptor ligation for LSU rRNA were primarily located at the 5’ end and were frequently distributed in the middle (S4 Fig), suggesting a breakdown at the middle position when handling RNA. Therefore, LSU rRNA formed at least one fragment with a similar size to SSU rRNA with a ligated adaptor and was the origin of the LSU rRNA sequences in the Pa-SSU dataset. Thus, considering the tedious step of SSU rRNA gel-extraction, the high quantity requirement of SSU rRNA and the low efficiency in removing LSU rRNA in the SSU rRNA enriched method, the total NA construction method may be more applicable than the former in low RNA yield samples that have low biomass, especially the interference of genomic DNA is confirmed to be absent. Therefore, the total NA method was used for the low biomass samples, such as tap water, shower curtain, leaf surfaces, mudflat water, and forehead (S2 Table).

### Coverage evaluation of 8F primer

Previous studies of 16S rRNA sequences have highlighted the necessity of accurate classification by employing near complete sequences [29, 41]. Primer 8F is generally used for bacterial community analysis with nearly full length 16S rRNA gene sequencing [42], and for analyzing the V1-V2 and V1-V3 regions of 16S rRNA gene sequences in next-generation sequencing [43–45]. The coverage of the bacterial universal primers for the 16S rRNA gene plays a crucial role in the correct understanding of microbial community structures.

In addition to information on microbial diversity, the position distribution of the 16S rRNA sequences also attracts the attention to the mismatches of the 8F primer. Although the non-coverage rates of 8F for bacteria were less than 3.5% in all datasets (Fig 4A), high non-coverage rates (up to 38.69%) were detected in unassigned sequences at the phylum level, which indicated the detection of many unidentified phyla-related sequences in these SSU rRNA reads. Therefore, we can design group specific primers based on 8F mismatch types in the phylum-unclassified sequences, and amplify nearly full length sequences that could be accurately assigned into novel candidate phyla [22, 46] paired with a universal reverse primer.

### Community structures for low microbial biomass environmental samples

The limitation for microbial population analysis that rely on the SSU rRNA-based sequence method is the difficulty in obtaining high quantities of enriched SSU rRNA from various environmental samples [11]. The total NA method was applied to different environmental samples with relatively low contents of total RNA (S2 Table) without DNase I treatment.

In drinking water, the physiological activity of the nitrogen cycle is of great importance to human health [47, 48]. Previously reported *Rhizobiales* [49] and *Comamonadacea* [50] related bacteria were also been detected with a relatively high potential activity in the present study. However, the RNA-seq data revealed that genus *Nitrospira* was also relatively abundant in the community rather than scarce as reported previously by the normal PCR amplification method [49, 51]. Members within this genus are important for nitrite oxidation and can even comprise as high as 78% of the biofilm forming cells in the distribution systems [52]. More interestingly, some bacteria within this group were discovered to be complete ammonia oxidizing bacteria [53, 54]. This suggested that the contribution of *Nitrospira* in nitrification of drinking water systems might have been previously underestimated by the general PCR method. Similar to *Nitrospira*, the phylum *Acidobacteria* was also an underestimated taxon by the PCR method compared with our RNA-seq data [49, 51]. Together with *Nitrospira*, this phylum was reported as an important participant in biofilm formation in drinking water systems and was unlikely to be of relevance to human health [49, 55, 56].

Opportunistic pathogens of the genera *Sphingomonas* and *Methylobacterium* in the α-*Proteobacteria* were once reported to dominate on shower curtains by 16S rRNA gene amplicon data [57, 58]. However, the 16S rRNA data in this study revealed that the phylum *Actinobacteria* was the most important. Its prevalent potentially active members, including those in the families *Intrasporangiaceae* (22.5%), *Micrococcaceae* (19.0%) and *Nocardiaceae* (12.3%) (S6 Table), were all reported to include increasing number of human-related pathogens [59–61].

The genera *Sphingomonas* and *Methylobacterium* were also the most common microbial community members of plant leaf surfaces and have great importance to plant health [62]. However, in the current RNA-seq dataset, their dominances were replaced by the phylum *Cyanobacteria* (S6 Table), which has been rarely reported before in the phyllosphere [63–66]. Members of this phylum are important primary producers and play important roles in the global carbon, oxygen and nitrogen cycles [67]. Notably, the potentially active unicellular genus *Chroococcidiopsis* was detected by our method, and members of this genus may contribute to the fixation of atmospheric nitrogen [68]. For the plant they inhabited, the acquisition of nitrogen from phyllosphere-associated cyanobacteria is an important mechanism [63].

*Eukaryota*, especially filamentous fungi and yeasts, was also a major group on the leaf surfaces, though bacteria dominated the microbial inhabitants [69, 70]. In contrast, approximately 66.3% of the SSU rRNA sequences in the RNA-seq dataset were assigned to fungi and mostly belonged to the phylum *Ascomycota* (S6 Table). Unlike the previous consensus, the prevalent orders *Dothideales* and *Pleosporales* were predominated by undescribed microbes with an unknown ecological function. Therefore, it is believed that their detailed classification requires further efforts. In addition, their underestimated potential roles also require further research because of their relevance to plant health.

Due to scarce data, the microbial community within mudflat surface water and the fungal community on the shower curtain cannot be directly compared with our RNA-seq data. However, the potentially active microbes revealed by our method are believed to be of great importance to the *in situ* physiological activity. This is also evidenced by the dominance of the potentially active genera *Propionibacterium* and *Malassezia* in the forehead data (S6 Table). Both genera are more related to human health, and their dominance here is also consistent with previous results obtained using PCR amplicons [71–73].

Therefore, the modified method is a powerful tool for characterizing potentially active microbial communities. Upon its broad application in microbial ecological analysis, it will also likely help in the discovery of more novel potentially active taxa on the basis of direct datasets of 8F primer mismatches it supplied.

## Conclusions

In this study, a modified RNA-seq library preparation method that was suitable for SSU rRNA-based community analysis was developed and tested. The accuracy of the method was confirmed by the analysis of three mock communities. By pre-ligating the adaptor to the 5’ end of RNA, it is feasible to prepare RNA-seq libraries from total nucleic acids of environmental samples, even those with low quantities of total RNA, without elimination of DNA. Another advantage is the characterization of the diversity of microbial communities by the OTU-based method and the increased efficiency of generating 16S rRNA sequences for OTU analysis, even compared with our previous study [20]. Since high non-coverage rates of the bacterial ‘universal’ primer 8F for unassigned sequences at phylum level were determined in this study, the novel taxa-specific modified 8F primer and a reverse universal primer (e.g. 1492R) can be used to amplify nearly full-length 16S rRNA gene sequences for accurately identifying novel bacterial lineages [22, 46].

The modified RNA-seq method in this study may be useful to determine potentially active microbial community structure and diversity for various environmental samples, and will also have use in the identification of novel microbial taxa.

## Supporting information

S1 Table Theoretical ratios and SSU rRNA quantities utilized in different mock communities for each isolate. (DOCX)

S2 Table Summary of obtained and utilized RNA quantities. (DOCX)

S3 Table Number of sequences generated from paired-end sequencing of RNA-seq libraries for mock communities. (DOCX)

S4 Table Determined SSU rRNA ratios of five reference strains in mock communities after RNA-seq analysis by different quality controls of sequence lengths. (DOCX)

S5 Table Non-coverage rates of the bacterial 8F primer at different taxonomic levels in the RNA-seq datasets of environmental samples. The taxa with a non-coverage sequence number of less than 10 in the 16S rRNA data are not shown. Numbers in the brackets to the left of slashes denotes non-coverage sequences of the taxa, while numbers to the right of slashes denotes total sequences classified into the taxa. A single mismatch in a sequence was not allowed. The non-coverage rate for a taxon was calculated by dividing its non-coverage sequences with its total sequences. Explanations for abbreviations are given in Table 1.
(XLSX)

S6 Table Bacterial and eukaryotic communities of the five low-biomass samples. Eukaryotic taxa with relative abundances <1% are not displayed. Explanations for abbreviations are given in Table 1.
(XLSX)

S7 Table Archaeal and bacterial richness estimation at different distance cut-off values for different environmental samples. Explanations for abbreviations are given in Table 1.
(DOCX)

S1 Fig Comparison of prokaryotic community structures between the OTU-based method and all SSU rRNA–based method for total NA derived dataset. Comparison of the community structures between the OTU-based method and all SSU rRNA -based method at different taxonomic levels (a Phylum, b Class, c Order, d Family, e Genus). The percentages calculated by the two methods were plotted, and the values in the legends are the slopes and coefficients of the regression equations, respectively. The explanation for Pa-Total is given in Table 1.
(TIF)

S2 Fig Comparison of prokaryotic community structures between the OTU-based method and all SSU rRNA–based method for gel-extracted SSU rRNA derived dataset. Comparison of the community structures between the OTU-based method and all SSU rRNA-based method at different taxonomic levels (a phylum, b class, c order, d family, e genus). The percentages calculated by the two methods were plotted, and the values in the legends are the slopes and coefficients of the regression equations, respectively. The explanation for Pa-SSU is given in Table 1.
(TIF)

S3 Fig Archaeal and bacterial richness of different environmental samples. Diversity index values were calculated using randomly selected sequences; for archaea, 500 sequences were randomly selected; for bacteria, 5000 sequences were randomly selected. ar, archaea; ba, bacteria. Explanations for abbreviations are given in Table 1.
(TIF)

S4 Fig Starting point distribution of LSU rRNA sequences for the five isolates in the Mock 2 community. A comparison was conducted between different RNA processing methods, without (blue) and with (pink) RNA denaturation. The x-axis indicates the position of first nucleotide in the LSU rRNA sequences that ligated to adaptor, and the y-axis indicates the percentages in all LSU rRNA sequences obtained.
(TIF)

## References

[1] Schmidt TM, DeLong EF, Pace NR. Analysis of a marine picoplankton community by 16S rRNA gene cloning and sequencing. J Bacteriol. 1991;173:4371–4378. 2066334 10.1128/jb.173.14.4371-4378.1991 PMC208098

[2] Muyzer G, de Waal EC, Uitterlinden AG. Profiling of complex microbial populations by denaturing gradient gel electrophoresis analysis of polymerase chain reaction-amplified genes coding for 16S rRNA. Appl Environ Microbiol. 1993;59:695–700. 7683183 10.1128/aem.59.3.695-700.1993 PMC202176

[3] Liu WT, Marsh TL, Cheng H, Forney LJ. Characterization of microbial diversity by determining terminal restriction fragment length polymorphisms of genes encoding 16S rRNA. Appl Environ Microbiol. 1997;63:4516–4522. 9361437 10.1128/aem.63.11.4516-4522.1997 PMC168770

[4] Shendure J, Ji H. Next-generation DNA sequencing. Nat Biotechnol. 2008;26(10):1135–1145. doi: 10.1038/nbt1486 18846087 10.1038/nbt1486

[5] Urich T, Lanzen A, Qi J, Huson DH, Schleper C, Schuster SC. Simultaneous assessment of soil microbial community structure and function through analysis of the meta-transcriptome. PLoS One. 2008;3:e2527 doi: 10.1371/journal.pone.0002527 18575584 10.1371/journal.pone.0002527 PMC2424134

[6] Klindworth A, Pruesse E, Schweer T, Peplies J, Quast C, Horn M, et al Evaluation of general 16S ribosomal RNA gene PCR primers for classical and next-generation sequencing-based diversity studies. Nucleic Acids Res. 2012;41(1):e1 doi: 10.1093/nar/gks808 22933715 10.1093/nar/gks808 PMC3592464

[7] Suzuki MT, Giovannoni SJ. Bias caused by template annealing in the amplification of mixtures of 16S rRNA genes by PCR. Appl Environ Microbiol. 1996;62(2):625–630. 8593063 10.1128/aem.62.2.625-630.1996 PMC167828

[8] Mao DP, Zhou Q, Chen CY, Quan ZX. Coverage evaluation of universal bacterial primers using the metagenomic datasets. BMC Microbiol. 2012;12:66 doi: 10.1186/1471-2180-12-66 22554309 10.1186/1471-2180-12-66 PMC3445835

[9] Tremblay J, Singh K, Fern A, Kirton ES, He S, Woyke T, et al Primer and platform effects on 16S rRNA tag sequencing. Front Microbiol. 2015;6:771 doi: 10.3389/fmicb.2015.00771 26300854 10.3389/fmicb.2015.00771 PMC4523815

[10] Mou X, Sun S, Edwards RA, Hodson RE, Moran MA. Bacterial carbon processing by generalist species in the coastal ocean. Nature. 2008;451(7179):708–711. doi: 10.1038/nature06513 18223640 10.1038/nature06513

[11] McDonald JE, Larsen N, Pennington A, Connolly J, Wallis C, Rooks DJ, et al Characterising the canine oral microbiome by direct sequencing of reverse-transcribed rRNA molecules. PLoS One. 2016;11:e157046. 10.1371/journal.pone.0157046 PMC4898712 27276347

[12] Rosselli R, Romoli O, Vitulo N, Vezzi A, Campanaro S, de Pascale F, et al Direct 16S rRNA-seq from bacterial communities: a PCR-independent approach to simultaneously assess microbial diversity and functional activity potential of each taxon. Sci Rep. 2016;6:32165 doi: 10.1038/srep32165 27577787 10.1038/srep32165 PMC5006002

[13] Logares R, Sunagawa S, Salazar G, Cornejo-Castillo FM, Ferrera I, Sarmento H, et al Metagenomic 16S rDNA Illumina tags are a powerful alternative to amplicon sequencing to explore diversity and structure of microbial communities. Environ Microbiol. 2014;16(9):2659–2671. doi: 10.1111/1462-2920.12250 24102695 10.1111/1462-2920.12250

[14] He S, Wurtzel O, Singh K, Froula JL, Yilmaz S, Tringe SG, et al Validation of two ribosomal RNA removal methods for microbial metatranscriptomics. Nat Methods. 2010;7:807–812. doi: 10.1038/nmeth.1507 20852648 10.1038/nmeth.1507

[15] Frias-Lopez J, Shi Y, Tyson GW, Coleman ML, Schuster SC, Chisholm SW, et al Microbial community gene expression in ocean surface waters. Proc Natl Acad Sci USA. 2008;105:3805–3810. doi: 10.1073/pnas.0708897105 18316740 10.1073/pnas.0708897105 PMC2268829

[16] Gilbert JA, Field D, Huang Y, Edwards R, Li W, Gilna P, et al Detection of large numbers of novel sequences in the metatranscriptomes of complex marine microbial communities. PLoS One. 2008;3:e3042 doi: 10.1371/journal.pone.0003042 18725995 10.1371/journal.pone.0003042 PMC2518522

[17] Yu K, Zhang T. Metagenomic and metatranscriptomic analysis of microbial community structure and gene expression of activated sludge. PLoS One. 2012;7(5):e38183 doi: 10.1371/journal.pone.0038183 22666477 10.1371/journal.pone.0038183 PMC3364235

[18] Tveit AT, Urich T, Svenning MM. Metatranscriptomic analysis of arctic peat soil microbiota. Appl Environ Microbiol. 2014;80:5761–5772. doi: 10.1128/AEM.01030-14 25015892 10.1128/AEM.01030-14 PMC4178616

[19] Tveit AT, Urich T, Frenzel P, Svenning MM. Metabolic and trophic interactions modulate methane production by Arctic peat microbiota in response to warming. Proc Natl Acad Sci USA. 2015;112:E2507–E2516. doi: 10.1073/pnas.1420797112 25918393 10.1073/pnas.1420797112 PMC4434766

[20] Li XR, Lv Y, Meng H, Gu JD, Quan ZX. Analysis of microbial diversity by pyrosequencing the small-subunit ribosomal RNA without PCR amplification. Appl Microbiol Biot. 2014;98:3777–3789. 10.1007/s00253-014-5583-0 24531274

[21] Blazewicz SJ, Barnard RL, Daly RA, Firestone MK. Evaluating rRNA as an indicator of microbial activity in environmental communities: limitations and uses. ISME J. 2013;7(11):2061–2068. doi: 10.1038/ismej.2013.102 23823491 10.1038/ismej.2013.102 PMC3806256

[22] Youssef NH, Couger MB, McCully AL, Criado AE, Elshahed MS. Assessing the global phylum level diversity within the bacterial domain: A review. J Adv Res. 2015;6:269–282. doi: 10.1016/j.jare.2014.10.005 26257925 10.1016/j.jare.2014.10.005 PMC4522544

[23] Zeleke J, Sheng Q, Wang JG, Huang MY, Xia F, Wu JH, et al Effects of *Spartina alterniflora* invasion on the communities of methanogens and sulfate-reducing bacteria in estuarine marsh sediments. Front Microbiol. 2013;4:243 doi: 10.3389/fmicb.2013.00243 23986751 10.3389/fmicb.2013.00243 PMC3750361

[24] Amin-ul Mannan M, Sharma S, Ganesan K. Total RNA isolation from recalcitrant yeast cells. Anal Biochem. 2009;389:77–79. doi: 10.1016/j.ab.2009.03.014 19302974 10.1016/j.ab.2009.03.014

[25] Lüdemann H, Arth I, Liesack W. Spatial changes in the bacterial community structure along a vertical oxygen gradient in flooded paddy soil cores. Appl Environ Microbiol. 2000;66:754–762. 10653747 10.1128/aem.66.2.754-762.2000 PMC91892

[26] Joshi NA, Fass JN. Sickle: A sliding-window, adaptive, quality-based trimming tool for FastQ files (Version 1.33). 2011. Available at https://github.com/najoshi/sickle.

[27] Caporaso JG, Kuczynski J, Stombaugh J, Bittinger K, Bushman FD, Costello EK, et al QIIME allows analysis of high-throughput community sequencing data. Nat Methods. 2010;7:335–336. doi: 10.1038/nmeth.f.303 20383131 10.1038/nmeth.f.303 PMC3156573

[28] Schloss PD, Westcott SL, Ryabin T, Hall JR, Hartmann M, Hollister EB, et al Introducing mothur: open-source, platform-independent, community-supported software for describing and comparing microbial communities. Appl Environ Microbiol. 2009;75:7537–7541. doi: 10.1128/AEM.01541-09 19801464 10.1128/AEM.01541-09 PMC2786419

[29] Wang Q, Garrity GM, Tiedje JM, Cole JR. Naive Bayesian classifier for rapid assignment of rRNA sequences into the new bacterial taxonomy. Appl Environ Microbiol. 2007;73:5261–5267. doi: 10.1128/AEM.00062-07 17586664 10.1128/AEM.00062-07 PMC1950982

[30] Kozich JJ, Westcott SL, Baxter NT, Highlander SK, Schloss PD. Development of a dual-index sequencing strategy and curation pipeline for analyzing amplicon sequence data on the MiSeq Illumina sequencing platform. Appl Environ Microbiol. 2013;79:5112–5120. doi: 10.1128/AEM.01043-13 23793624 10.1128/AEM.01043-13 PMC3753973

[31] Parks DH, Beiko RG. Identifying biologically relevant differences between metagenomic communities. Bioinformatics. 2010;26:715–721. doi: 10.1093/bioinformatics/btq041 20130030 10.1093/bioinformatics/btq041

[32] Walsh PS, Erlich HA, Higuchi R. Preferential PCR amplification of alleles: mechanisms and solutions. Genome Res. 1992;1(4):241–250. 10.1101/gr.1.4.241 1477658

[33] Zifcakova L, Vetrovsky T, Howe A, Baldrian P. Microbial activity in forest soil reflects the changes in ecosystem properties between summer and winter. Environ Microbiol. 2016;18:288–301. doi: 10.1111/1462-2920.13026 26286355 10.1111/1462-2920.13026

[34] Xiong X, Frank DN, Robertson CE, Hung SS, Markle J, Canty AJ, et al Generation and analysis of a mouse intestinal metatranscriptome through Illumina based RNA-sequencing. PLoS One. 2012;7(4):e36009 doi: 10.1371/journal.pone.0036009 22558305 10.1371/journal.pone.0036009 PMC3338770

[35] van Dijk EL, Jaszczyszyn Y, Thermes C. Library preparation methods for next-generation sequencing: tone down the bias. Exp Cell Res. 2014;322:12–20. doi: 10.1016/j.yexcr.2014.01.008 24440557 10.1016/j.yexcr.2014.01.008

[36] Hansen KD, Brenner SE, Dudoit S. Biases in Illumina transcriptome sequencing caused by random hexamer priming. Nucleic Acids Res. 2010;38:e131 doi: 10.1093/nar/gkq224 20395217 10.1093/nar/gkq224 PMC2896536

[37] Hafner M, Renwick N, Brown M, Mihailovic A, Holoch D, Lin C, et al RNA-ligase-dependent biases in miRNA representation in deep-sequenced small RNA cDNA libraries. RNA. 2011;17(9):1697–1712. doi: 10.1261/rna.2799511 21775473 10.1261/rna.2799511 PMC3162335

[38] Wintzingerode FV, Göbel UB, Stackebrandt E. Determination of microbial diversity in environmental samples: pitfalls of PCR-based rRNA analysis. FEMS Microbiol Rev. 1997;21(3):213–229. 9451814 10.1111/j.1574-6976.1997.tb00351.x

[39] Weller R, Ward DM. Selective recovery of 16S rRNA sequences from natural microbial communities in the form of cDNA. Appl Environ Microbiol. 1989;55:1818–1822. 16347975 10.1128/aem.55.7.1818-1822.1989 PMC202956

[40] Botero LM, D'Imperio S, Burr M, McDermott TR, Young M, Hassett DJ. Poly(A) polymerase modification and reverse transcriptase PCR amplification of environmental RNA. Appl Environ Microbiol. 2005;71:1267–1275. doi: 10.1128/AEM.71.3.1267-1275.2005 15746328 10.1128/AEM.71.3.1267-1275.2005 PMC1065135

[41] Yarza P, Yilmaz P, Pruesse E, Glockner FO, Ludwig W, Schleifer KH, et al Uniting the classification of cultured and uncultured bacteria and archaea using 16S rRNA gene sequences. Nat Rev Microbiol. 2014;12:635–645. doi: 10.1038/nrmicro3330 25118885 10.1038/nrmicro3330

[42] Frank JA, Reich CI, Sharma S, Weisbaum JS, Wilson BA, Olsen GJ. Critical evaluation of two primers commonly used for amplification of bacterial 16S rRNA genes. Appl Environ Microbiol. 2008;74(8):2461–2470. doi: 10.1128/AEM.02272-07 18296538 10.1128/AEM.02272-07 PMC2293150

[43] Youssef N, Sheik CS, Krumholz LR, Najar FZ, Roe BA, Elshahed MS. Comparison of species richness estimates obtained using nearly complete fragments and simulated pyrosequencing-generated fragments in 16S rRNA gene-based environmental surveys. Appl Environ Microbiol. 2009;75(16):5227–5236. doi: 10.1128/AEM.00592-09 19561178 10.1128/AEM.00592-09 PMC2725448

[44] Salipante SJ, Kawashima T, Rosenthal C, Hoogestraat DR, Cummings LA, Sengupta DJ, et al Performance comparison of Illumina and ion torrent next-generation sequencing platforms for 16S rRNA-based bacterial community profiling. Appl Environ Microbiol. 2014;80(24):7583–7591. doi: 10.1128/AEM.02206-14 25261520 10.1128/AEM.02206-14 PMC4249215

[45] Kumar PS, Brooker MR, Dowd SE, Camerlengo T. Target region selection is a critical determinant of community fingerprints generated by 16S pyrosequencing. PLoS One. 2011;6(6):e20956 doi: 10.1371/journal.pone.0020956 21738596 10.1371/journal.pone.0020956 PMC3126800

[46] Youssef N, Steidley BL, Elshahed MS. Novel high-rank phylogenetic lineages within a sulfur spring (Zodletone Spring, Oklahoma), revealed using a combined pyrosequencing-sanger approach. Appl Environ Microbiol. 2012;78:2677–2688. doi: 10.1128/AEM.00002-12 22307312 10.1128/AEM.00002-12 PMC3318811

[47] Zhang Y, Love N, Edwards M. Nitrification in drinking water systems. Crit Rev Env Sci Tec. 2009;39(3):153–208.

[48] Mizuta K, Matsumoto T, Hatate Y, Nishihara K, Nakanishi T. Removal of nitrate-nitrogen from drinking water using bamboo powder charcoal. Bioresource Technol. 2004;95:255–257. 10.1016/j.biortech.2004.02.015 15288267

[49] Henne K, Kahlisch L, Brettar I, Hofle MG. Analysis of structure and composition of bacterial core communities in mature drinking water biofilms and bulk water of a citywide network in Germany. Appl Environ Microbiol. 2012;78(10):3530–3538. doi: 10.1128/AEM.06373-11 22389373 10.1128/AEM.06373-11 PMC3346358

[50] Pinto AJ, Schroeder J, Lunn M, Sloan W, Raskin L. Spatial-temporal survey and occupancy-abundance modeling to predict bacterial community dynamics in the drinking water microbiome. mBio. 2014;5(3):e1114–e1135. 10.1128/mBio.01135-14 PMC4045074 24865557

[51] Holinger EP, Ross KA, Robertson CE, Stevens MJ, Harris JK, Pace NR. Molecular analysis of point-of-use municipal drinking water microbiology. Water Res. 2014;49:225–235. doi: 10.1016/j.watres.2013.11.027 24333849 10.1016/j.watres.2013.11.027

[52] Berry D, Xi C, Raskin L. Microbial ecology of drinking water distribution systems. Curr Opin Biotech. 2006;17(3):297–302. doi: 10.1016/j.copbio.2006.05.007 16701992 10.1016/j.copbio.2006.05.007

[53] Daims H, Lebedeva EV, Pjevac P, Han P, Herbold C, Albertsen M, et al Complete nitrification by *Nitrospira* bacteria. Nature. 2015;528:504–509. doi: 10.1038/nature16461 26610024 10.1038/nature16461 PMC5152751

[54] van Kessel MA, Speth DR, Albertsen M, Nielsen PH, Op Den Camp HJ, Kartal B, et al Complete nitrification by a single microorganism. Nature. 2015;528:555–559. doi: 10.1038/nature16459 26610025 10.1038/nature16459 PMC4878690

[55] Eichler S, Christen R, Höltje C, Westphal P, Bötel J, Brettar I, et al Composition and dynamics of bacterial communities of a drinking water supply system as assessed by RNA-and DNA-based 16S rRNA gene fingerprinting. Appl Environ Microbiol. 2006;72(3):1858–1872. doi: 10.1128/AEM.72.3.1858-1872.2006 16517632 10.1128/AEM.72.3.1858-1872.2006 PMC1393179

[56] Martiny AC, Albrechtsen H, Arvin E, Molin S. Identification of bacteria in biofilm and bulk water samples from a nonchlorinated model drinking water distribution system: detection of a large nitrite-oxidizing population associated with *Nitrospira* spp. Appl Environ Microbiol. 2005;71(12):8611–8617. doi: 10.1128/AEM.71.12.8611-8617.2005 16332854 10.1128/AEM.71.12.8611-8617.2005 PMC1317318

[57] Kelley ST, Theisen U, Angenent LT, St. Amand A, Pace NR. Molecular analysis of shower curtain biofilm microbes. Appl Environ Microb. 2004;70(7):4187–4192. 10.1128/AEM.70.7.4187-4192.2004 PMC444822 15240300

[58] Kelley ST, Gilbert JA. Studying the microbiology of the indoor environment. Genome Biol. 2013;14:202 doi: 10.1186/gb-2013-14-2-202 23514020 10.1186/gb-2013-14-2-202 PMC3663111

[59] Goodfellow M. The Family *Nocardiaceae* In: Rosenberg E, DeLong EF, Lory S, Stackebrandt E, Thompson F, editors. The Prokaryotes. Berlin: Springer; 2014 p. 595–650.

[60] Stackebrandt E, Scheuner C, Göker M, Schumann P. The Family *Intrasporangiaceae* In: Rosenberg E, DeLong EF, Lory S, Stackebrandt E, Thompson F, editors. The Prokaryotes. Berlin: Springer; 2014 p. 397–424.

[61] Dastager SG, Krishnamurthi S, Rameshkumar N, Dharne M. The Family *Micrococcaceae* In: Rosenberg E, DeLong EF, Lory S, Stackebrandt E, Thompson F, editors. The Prokaryotes. Berlin: Springer; 2014 p. 455–498.

[62] Bulgarelli D, Schlaeppi K, Spaepen S, Ver Loren Van Themaat E, Schulze-Lefert P. Structure and functions of the bacterial microbiota of plants. Annu Rev Plant Biol. 2013;64:807–838. doi: 10.1146/annurev-arplant-050312-120106 23373698 10.1146/annurev-arplant-050312-120106

[63] Rigonato J, Gonçalves N, Andreote APD, Lambais MR, Fiore MF. Estimating genetic structure and diversity of cyanobacterial communities in Atlantic forest phyllosphere. Can J Microbiol. 2016:1–8. 10.1139/cjm-2016-0229 27696898

[64] Venkatachalam S, Ranjan K, Prasanna R, Ramakrishnan B, Thapa S, Kanchan A. Diversity and functional traits of culturable microbiome members, including cyanobacteria in the rice phyllosphere. Plant Biol. 2016;18(4):627–637. doi: 10.1111/plb.12441 26849835 10.1111/plb.12441

[65] Rigonato J, Alvarenga DO, Andreote FD, Dias ACF, Melo IS, Kent A, et al Cyanobacterial diversity in the phyllosphere of a mangrove forest. FEMS Microbiol Ecol. 2012;80(2):312–322. 22611551 10.1111/j.1574-6941.2012.01299.x

[66] Redford AJ, Bowers RM, Knight R, Linhart Y, Fierer N. The ecology of the phyllosphere: geographic and phylogenetic variability in the distribution of bacteria on tree leaves. Environ Microbiol. 2010;12:2885–2893. doi: 10.1111/j.1462-2920.2010.02258.x 20545741 10.1111/j.1462-2920.2010.02258.x PMC3156554

[67] Tomitani A, Knoll AH, Cavanaugh CM, Ohno T. The evolutionary diversification of cyanobacteria: molecular–phylogenetic and paleontological perspectives. Proc Natl Acad Sci USA. 2006;103(14):5442–5447. doi: 10.1073/pnas.0600999103 16569695 10.1073/pnas.0600999103 PMC1459374

[68] Dojani S, Lakatos M, Rascher U, Wanek W, Lüttge U, Büdel B. Nitrogen input by cyanobacterial biofilms of an inselberg into a tropical rainforest in French Guiana. Flora. 2007;202(7):521–529.

[69] Whipps JM, Hand P, Pink D, Bending GD. Phyllosphere microbiology with special reference to diversity and plant genotype. J Appl Microbiol. 2008;105(6):1744–1755. doi: 10.1111/j.1365-2672.2008.03906.x 19120625 10.1111/j.1365-2672.2008.03906.x

[70] Vorholt JA. Microbial life in the phyllosphere. Nat Rev Microbiol. 2012;10(12):828–840. doi: 10.1038/nrmicro2910 23154261 10.1038/nrmicro2910

[71] Grice EA, Kong HH, Conlan S, Deming CB, Davis J, Young AC, et al Topographical and temporal diversity of the human skin microbiome. Science. 2009;324(5931):1190–1192. doi: 10.1126/science.1171700 19478181 10.1126/science.1171700 PMC2805064

[72] Grice EA, Segre JA. The skin microbiome. Nat Rev Microbiol. 2011;9:244–253. doi: 10.1038/nrmicro2537 21407241 10.1038/nrmicro2537 PMC3535073

[73] Adams RI, Miletto M, Taylor JW, Bruns TD. The diversity and distribution of fungi on residential surfaces. PLoS One. 2013;8(11):e78866 doi: 10.1371/journal.pone.0078866 24223861 10.1371/journal.pone.0078866 PMC3815347

