# Supplementary material for: Modified RNA-seq method for microbial community and diversity analysis using rRNA in different types of environmental samples
Source: PLoS One. 2017 Oct 10;12(10):e0186161. doi: 10.1371/journal.pone.0186161 (PMC5634646; doi:10.1371/journal.pone.0186161)
Supplement: S1 Table — (DOCX) [file pone.0186161.s001.DOCX]

**S1 Table. Theoretical ratios and SSU rRNA quantities utilized in different mock communities**

**for each isolate.**

|  | **Mock 1** | | | **Mock 2** | | | **Mock 3** | | |
| --- | --- | --- | --- | --- | --- | --- | --- | --- | --- |
|  | **Ratios** | **1^a^** | **2^b^** | **Ratios** | **1** | **2** | **Ratios** | **1** | **2** |
| ***B.subtilis*** | 0.1 | 0.1 | 0.1 | 1 | 2 | 3 | 10 | 10 | 10 |
| ***C.caeni*** | 10 | 10 | 10 | 1 | 2 | 3 | 0.1 | 0.1 | 0.1 |
| ***E.coli*** | 1 | 1 | 1 | 1 | 2 | 3 | 1 | 1 | 1 |
| ***H.halobium*** | 0.1 | 0.1 | 0.1 | 1 | 2 | 3 | 10 | 10 | 10 |
| ***P.pastoris*** | 10 | 10 | 10 | 1 | 2 | 3 | 0.1 | 0.1 | 0.1 |
| **Total quantity (ng)** |  | 21.2 | 21.2 |  | 10.0 | 15 |  | 21.2 | 21.2 |

^a^ Quantity of SSU rRNA that was utilized for library construction without RNA denaturation

prior to adaptor ligation.

^b^ Quantity of SSU rRNA that was utilized for library construction with RNA denaturation prior

to adaptor ligation.
