# Supplementary material for: Modified RNA-seq method for microbial community and diversity analysis using rRNA in different types of environmental samples
Source: PLoS One. 2017 Oct 10;12(10):e0186161. doi: 10.1371/journal.pone.0186161 (PMC5634646; doi:10.1371/journal.pone.0186161)
Supplement: S2 Table — (DOCX) [file pone.0186161.s002.DOCX]

**S2 Table. Summary of the obtained and utilized RNA quantities.**

| **Sample names** | **Extracted concentration (ng/μL)** | **Total quantity (ng)** | **Quantity for RNA-seq**  **(ng)** |
| --- | --- | --- | --- |
| **Sa** ^a^ | 118.00 | 5900 | 25.00 |
| **Pa-Total** ^b^ | 92.00 | 4600 | 100.00 |
| **Pa-SSU** ^c^ | 6.64 | 79.68 | 25.00 |
| **Tap Water (TW) ^d^** | 2.50 | 250 | 20.00 |
| **Shower Curtain (SC) ^d^** | 4.16 | 416 | 20.80 |
| **Leaf Surface (LS) ^d^** | 1.80 | 180 | 18.00 |
| **Mudflat surface Water (MW) ^d^** | 12.40 | 1240 | 24.80 |
| **Forehead (FH) ^d^** | 0.80 | 80 | 16.00 |

^a^ Salt-marsh sediments vegetated by *Spartina alterniflora*, and total RNA after DNA removal was

quantified.

^b^ Salt-marsh sediments vegetated by *Phragmites australis*, and total RNA without DNA removal

was quantified.

^c^ Salt-marsh sediments vegetated by *Phragmites australis*, and enriched SSU rRNA by gel-extraction

was quantified.

^d^ Low-biomass samples, and total RNA without DNA removal was quantified.
