# Supplementary material for: Modified RNA-seq method for microbial community and diversity analysis using rRNA in different types of environmental samples
Source: PLoS One. 2017 Oct 10;12(10):e0186161. doi: 10.1371/journal.pone.0186161 (PMC5634646; doi:10.1371/journal.pone.0186161)
Supplement: S3 Table — (DOCX) [file pone.0186161.s003.DOCX]

**S3 Table. Number of sequences generated from paired-end sequencing of RNA-seq libraries for mock communities.**

|  | **Without RNA denaturation** | | | **With RNA denaturation** | | |
| --- | --- | --- | --- | --- | --- | --- |
|  | **Mock 1** | **Mock 2** | **Mock 3** | **Mock 1** | **Mock 2** | **Mock 3** |
| **Sequences after assembly** | 813,772 | 748,818 | 627,422 | 207,479 | 240,868 | 145,723 |
| **Sequences after QC^a^** | 813,110 | 748,195 | 626,989 | 206,574 | 240,099 | 145,579 |
| **>200 bp** | 795,552 **^c^** | 717,200 | 606,772 | 195,534 | 216,250 | 129,263 |
| **LSU** | 61,379 **^d^** **(7.7%)^e^** | 64,935 **(9.1%)** | 75,966 **(12.5%)** | 6,747**(3.5%)** | 6,944**(3.2%)** | 4,734 **(3.7%)** |
| **SSU** | 716,043**(90.0%)** | 637,065 **(88.8%)** | 521,790 **(86.0%)** | 156,931**(80.3%)** | 186,159**(86.1%)** | 122,337**(94.6%)** |
| **Others^b^** | 18,130 **(2.3%)** | 15,200 **(2.1%)** | 9,016 **(1.5%)** | 31,856**(16.3%)** | 23,147**(10.7%)** | 2,192**(1.7%)** |
| **>250 bp** | 790470 | 708,607 | 602,834 | 132,866 | 132,160 | 116,606 |
| **LSU** | 60,905 **(7.7%)** | 64,299 **(9.1%)** | 75,437 **(12.5%)** | 5,834 **(4.4%)** | 5,284 **(4.0%)** | 3,955 **(3.3%)** |
| **SSU** | 712,043 **(90.1%)** | 630,246 **(88.9%)** | 519,154 **(86.1%)** | 100,176 **(75.4%)** | 109,761 **(83.1%)** | 113,920 **(95.2%)** |
| **Others** | 17,522 **(2.2%)** | 14,062 **(2.0%)** | 8,243 **(1.4%)** | 26,856 **(20.2%)** | 17,115 **(12.9%)** | 1,731 **( 1.5%)** |
| **>360 bp** | 365,690 | 335,356 | 335,235 | 52,412 | 46,400 | 60,149 |
| **LSU** | 29,815 **(8.2%)** | 27,902**(8.3%)** | 37,929**(11.3%)** | 2,061**(3.9%)** | 1,465**(3.2%)** | 1,566**(2.6%)** |
| **SSU** | 324,407 **(88.7%)** | 299,691**(89.4%)** | 293,259 **(87.5%)** | 39,261**(74.9%)** | 39,608**(85.4%)** | 57,993**(96.4%)** |
| **Others** | 11,468 **(3.1%)** | 7,763 **(2.3%)** | 4,047**(1.2%)** | 11,090**(21.2%)** | 5,327**(11.5%)** | 590**(1.0%)** |

^a^ High-quality sequences after removal of assembled sequences that contained any ambiguous nucleotide and homopolymers >8 nt.

^b^ All sequences classified as non-rRNA.

^c^ Total number of high-quality sequences longer than a specific length in the relative mock community.

^d^ Number of high-quality sequences classified as a specific type in the relative mock community.

^e^ Percentages of a specific type of sequence in the total sequences of this mock community.
