# Supplementary material for: Modified RNA-seq method for microbial community and diversity analysis using rRNA in different types of environmental samples
Source: PLoS One. 2017 Oct 10;12(10):e0186161. doi: 10.1371/journal.pone.0186161 (PMC5634646; doi:10.1371/journal.pone.0186161)
Supplement: S4 Table — (DOCX) [file pone.0186161.s004.DOCX]

**S4 Table. Determined SSU rRNA ratios of five reference strains in mock communities after RNA-seq analysis by different**

**quality controls of sequence lengths.**

|  |  | **Theoretical ratios** ^a^ | **Without RNA denaturation** | | | **With RNA denaturation** | | |
| --- | --- | --- | --- | --- | --- | --- | --- | --- |
|  |  |  | **>200** ^b^ | **>250** | **>360** | **>200** | **>250** | **>360** |
| **Mock1** | ***B. subtilis*** | **0.1** | 0.10 | 0.10 | 0.10 | 0.12 | 0.13 | 0.16 |
|  | ***C. caeni*** | **10** | 24.31 | 24.18 | 12.15 | 36.80 | 22.48 | 14.21 |
|  | ***E. coli*** | **1** | 1.00 | 1.00 | 1.00 | 1.00 | 1.00 | 1.00 |
|  | ***H. halobium*** | **0.1** | 0.04 | 0.04 | 0.03 | 0.31 | 0.32 | 0.22 |
|  | ***P. pastoris*** | **10** | 9.62 | 9.61 | 9.75 | 9.03 | 9.17 | 11.54 |
| **Mock 2** | ***B. subtilis*** | **1** | 1.36 | 1.36 | 1.26 | 1.77 | 2.00 | 2.09 |
|  | ***C. caeni*** | **1** | 2.93 | 2.88 | 1.49 | 9.18 | 4.96 | 2.48 |
|  | ***E. coli*** | **1** | 1.00 | 1.00 | 1.00 | 1.00 | 1.00 | 1.00 |
|  | ***H. halobium*** | **1** | 0.52 | 0.52 | 0.40 | 0.77 | 0.83 | 0.36 |
|  | ***P. pastoris*** | **1** | 1.10 | 1.10 | 1.16 | 1.20 | 1.26 | 1.25 |
| **Mock 3** | ***B. subtilis*** | **10** | 10.61 | 10.60 | 9.81 | 19.34 | 20.86 | 20.70 |
|  | ***C. caeni*** | **0.1** | 0.16 | 0.16 | 0.08 | 0.58 | 0.35 | 0.15 |
|  | ***E. coli*** | **1** | 1.00 | 1.00 | 1.00 | 1.00 | 1.00 | 1.00 |
|  | ***H. halobium*** | **10** | 3.73 | 3.73 | 2.85 | 6.22 | 6.39 | 3.00 |
|  | ***P. pastoris*** | **0.1** | 0.11 | 0.11 | 0.11 | 0.12 | 0.12 | 0.12 |

^a^ All the ratios were calculated by dividing the number of reads of the relative isolate with *E. coli* reads.

^b^ Sequence length cut-off values for the quality control of sequences during analyses of RNA-seq datasets.
