# Supplementary material for: Modified RNA-seq method for microbial community and diversity analysis using rRNA in different types of environmental samples
Source: PLoS One. 2017 Oct 10;12(10):e0186161. doi: 10.1371/journal.pone.0186161 (PMC5634646; doi:10.1371/journal.pone.0186161)
Supplement: S7 Table — Explanations for abbreviations are given in Table 1. (DOCX) [file pone.0186161.s007.DOCX]

**S7 Table. Archaeal and Bacterial richness estimation at different distance cut-off values for different**

**environmental samples.**

|  |  | **Pa-Total_ar** | **Pa-SSU_ar** | **Pa-Total_ba** | **Pa-SSU_ba** | **TW_ba** | **SC_ba** | **LS_ba** | **MW_ba** | **FH_ba** |
| --- | --- | --- | --- | --- | --- | --- | --- | --- | --- | --- |
| **Total SSU counts** | | 3,383 | 4,027 | 159,684 | 262,015 | 51,198 | 70,878 | 16,254 | 9,542 | 62,870 |
| **Sequences for calculation^a^** | | 992 | 1,617 | 74,725 | 156,736 | 23,414 | 31,520 | 7,967 | 5,230 | 33,789 |
| **Percentages in total (%)^b^** | | 29.3 | 40.2 | 46.8 | 59.8 | 45.7 | 44.5 | 49.0 | 54.8 | 53.7 |
| **OTUs** | **0.03** | 151 | 173 | 8,934 | 12,029 | 1,054 | 797 | 1,827 | 642 | 1,994 |
|  | **0.05** | 135 | 145 | 7,360 | 9,664 | 900 | 610 | 1,496 | 564 | 1,714 |
|  | **0.10** | 113 | 109 | 4,771 | 6,116 | 650 | 402 | 962 | 432 | 1,218 |
|  | **0.20** | 56 | 52 | 1,832 | 2,084 | 295 | 185 | 380 | 207 | 516 |
|  | **0.30** | 18 | 15 | 556 | 519 | 92 | 64 | 96 | 69 | 143 |
| **Chao** | **0.03** | 376 | 510 | 18,047 | 22,229 | 1,829 | 2,165 | 4,877 | 1,196 | 5,178 |
|  | **0.05** | 309 | 366 | 13,787 | 16,769 | 1,474 | 1,420 | 3,593 | 1,154 | 4,106 |
|  | **0.10** | 255 | 212 | 8,261 | 9,812 | 1,026 | 894 | 2,126 | 854 | 2,897 |
|  | **0.20** | 118 | 67 | 3,153 | 3181 | 521 | 340 | 659 | 381 | 980 |
|  | **0.30** | 24 | 26 | 1,146 | 759 | 140 | 94 | 137 | 116 | 277 |
| **ACE** | **0.03** | 480 | 837 | 25,626 | 28,623 | 2,225 | 4,237 | 8,952 | 1,877 | 10,223 |
|  | **0.05** | 415 | 602 | 18,691 | 20,797 | 1,731 | 2,569 | 6,508 | 1,635 | 7,190 |
|  | **0.10** | 400 | 299 | 10,746 | 12,094 | 1,256 | 1,586 | 3,199 | 1,172 | 4,473 |
|  | **0.20** | 161 | 71 | 4,030 | 3,905 | 623 | 549 | 797 | 450 | 1,210 |
|  | **0.30** | 51 | 29 | 2,067 | 921 | 164 | 93 | 132 | 102 | 511 |
| **Shannon** | **0.03** | 3.44 | 3.66 | 7.28 | 7.27 | 4.90 | 3.54 | 5.55 | 4.96 | 1.71 |
|  | **0.05** | 3.10 | 3.28 | 7.01 | 7.01 | 4.69 | 3.34 | 5.30 | 4.84 | 1.66 |
|  | **0.10** | 2.55 | 2.70 | 6.37 | 6.38 | 4.26 | 2.90 | 4.62 | 4.46 | 1.53 |
|  | **0.20** | 1.71 | 1.71 | 4.58 | 4.52 | 3.06 | 1.73 | 2.83 | 3.36 | 1.17 |
|  | **0.30** | 0.77 | 0.72 | 2.66 | 2.48 | 2.10 | 0.37 | 1.55 | 1.61 | 0.83 |

^a^ Sequences covered the 8F-V1-V2 region between *E. coli* position 8 and 242 were used for diversity index calculations.

^b^ Percentages of 8F-V1-V2 region-containing sequences in the relative total SSU rRNA sequences.
