# Supplementary figures and images for: Modified RNA-seq method for microbial community and diversity analysis using rRNA in different types of environmental samples
Source: PLoS One. 2017 Oct 10;12(10):e0186161. doi: 10.1371/journal.pone.0186161 (PMC5634646; doi:10.1371/journal.pone.0186161)

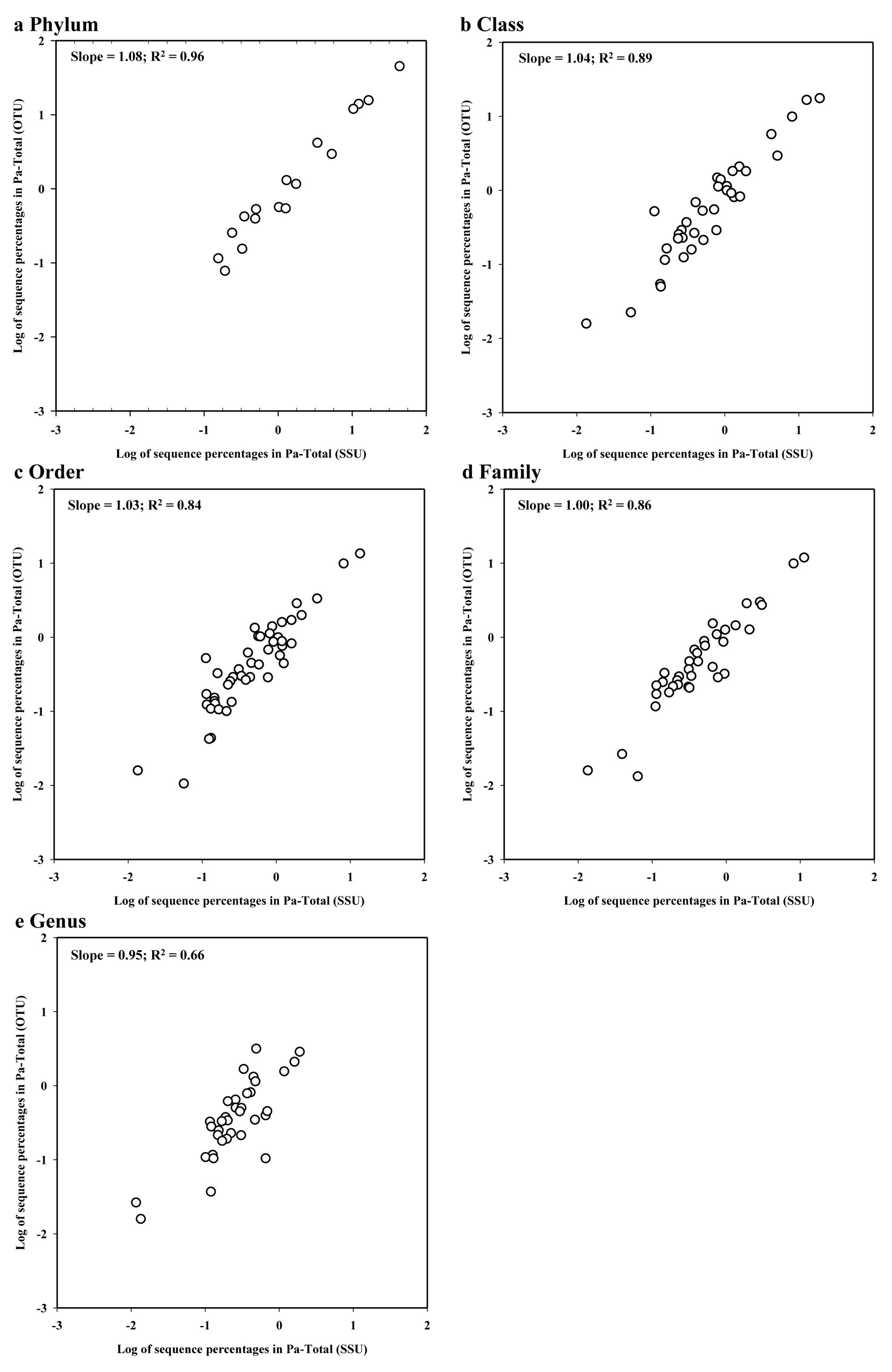

Supplement: S1 Fig — Comparison of the community structures between the OTU-based method and all SSU rRNA -based method at different taxonomic levels (a Phylum, b Class, c Order, d Family, e Genus). The percentages calculated by the two methods were plotted, and the values in the legends are the slopes and coefficients of the regression equations, respectively. The explanation for Pa-Total is given in Table 1. (TIF) [file pone.0186161.s008.TIF]

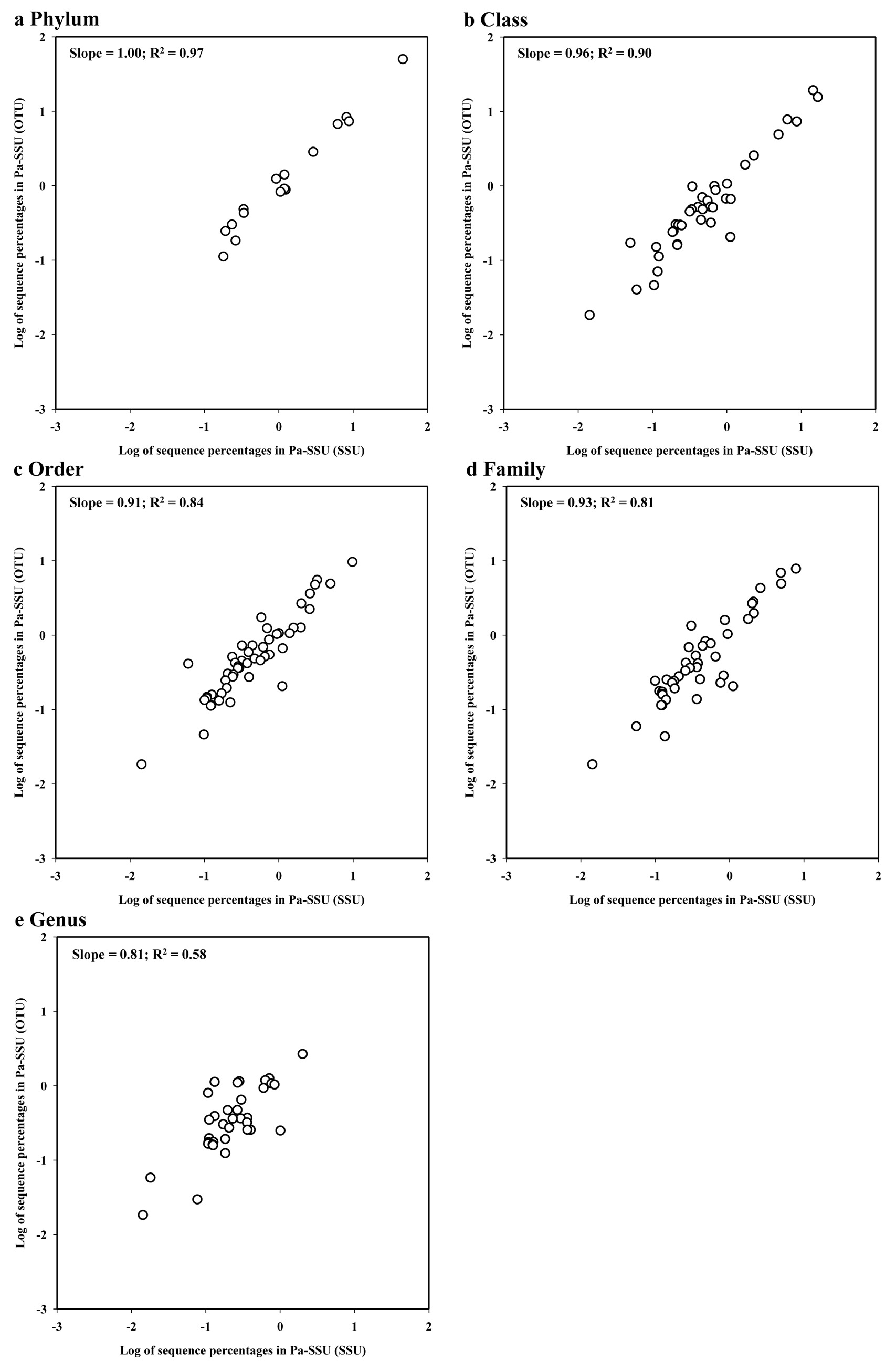

Supplement: S2 Fig — Comparison of the community structures between the OTU-based method and all SSU rRNA-based method at different taxonomic levels (a phylum, b class, c order, d family, e genus). The percentages calculated by the two methods were plotted, and the values in the legends are the slopes and coefficients of the regression equations, respectively. The explanation for Pa-SSU is given in Table 1. (TIF) [file pone.0186161.s009.TIF]

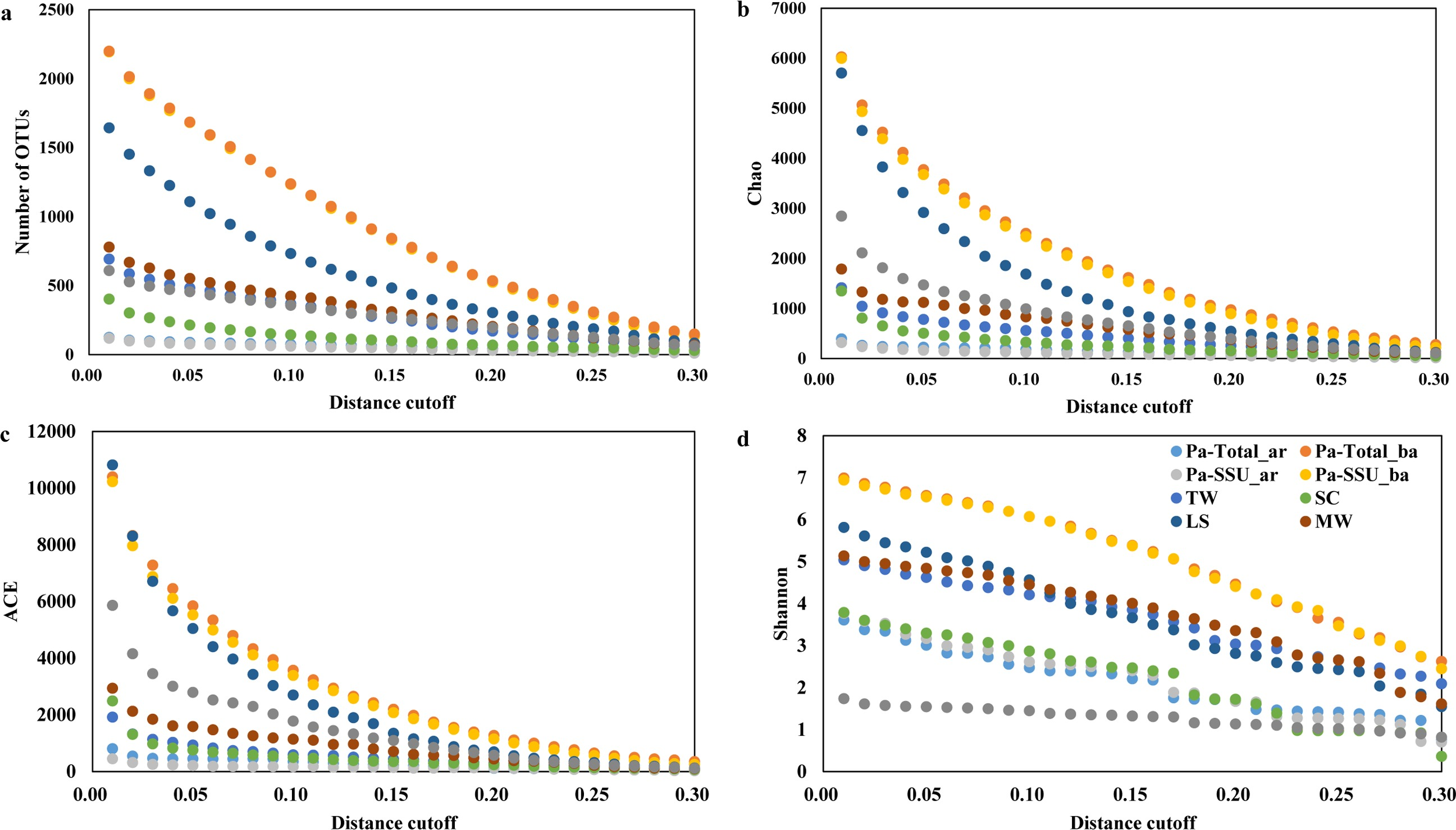

Supplement: S3 Fig — Diversity index values were calculated using randomly selected sequences; for archaea, 500 sequences were randomly selected; for bacteria, 5000 sequences were randomly selected. ar, archaea; ba, bacteria. Explanations for abbreviations are given in Table 1. (TIF) [file pone.0186161.s010.TIF]

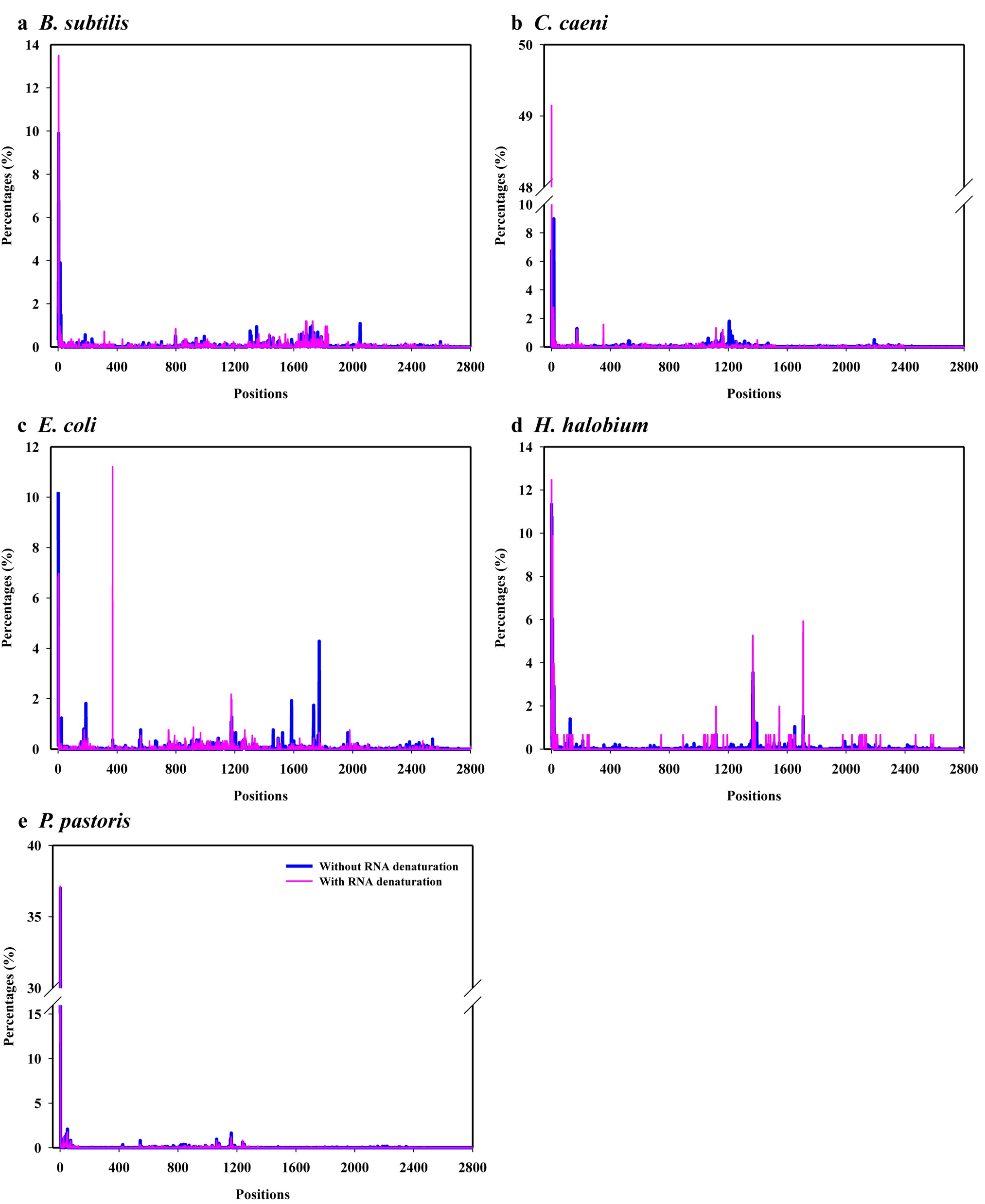

Supplement: S4 Fig — A comparison was conducted between different RNA processing methods, without (blue) and with (pink) RNA denaturation. The x-axis indicates the position of first nucleotide in the LSU rRNA sequences that ligated to adaptor, and the y-axis indicates the percentages in all LSU rRNA sequences obtained. (TIF) [file pone.0186161.s011.TIF]
